# Diphenylanthracene Dimers for Triplet–Triplet Annihilation Photon Upconversion: Mechanistic Insights for Intramolecular Pathways and the Importance of Molecular Geometry

**DOI:** 10.1021/jacs.1c00331

**Published:** 2021-04-09

**Authors:** Axel Olesund, Victor Gray, Jerker Mårtensson, Bo Albinsson

**Affiliations:** † Department of Chemistry and Chemical Engineering, Chalmers University of Technology, 412 96 Gothenburg, Sweden; ‡ Department of Chemistry, Ångström Laboratory, Uppsala University, Box 532, 751 20 Uppsala, Sweden

## Abstract

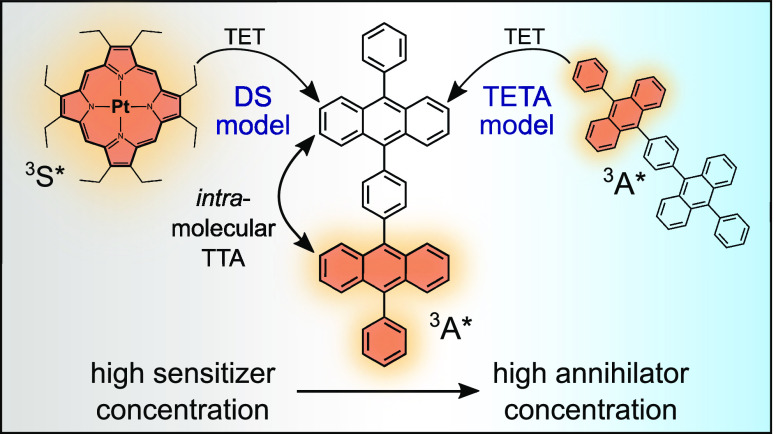

Novel approaches to modify the spectral output of the sun have seen a surge in interest recently, with triplet–triplet annihilation driven photon upconversion (TTA-UC) gaining widespread recognition due to its ability to function under low-intensity, noncoherent light. Herein, four diphenylanthracene (DPA) dimers are investigated to explore how the structure of these dimers affects upconversion efficiency. Also, the mechanism responsible for intramolecular upconversion is elucidated. In particular, two models are compared using steady-state and time-resolved simulations of the TTA-UC emission intensities and kinetics. All dimers perform TTA-UC efficiently in the presence of the sensitizer platinum octaethylporphyrin. The meta-coupled dimer 1,3-DPA_2_ performs best yielding a 21.2% upconversion quantum yield (out of a 50% maximum), which is close to that of the reference monomer DPA (24.0%). Its superior performance compared to the other dimers is primarily ascribed to the longer triplet lifetime of this dimer (4.7 ms), thus reinforcing the importance of this parameter. Comparisons between simulations and experiments reveal that the double-sensitization mechanism is part of the mechanism of intramolecular upconversion and that this additional pathway could be of great significance under specific conditions. The results from this study can thus act as a guide not only in terms of annihilator design but also for the design of future solid-state systems where intramolecular exciton migration is anticipated to play a major role.

## Introduction

**Figure 1:**
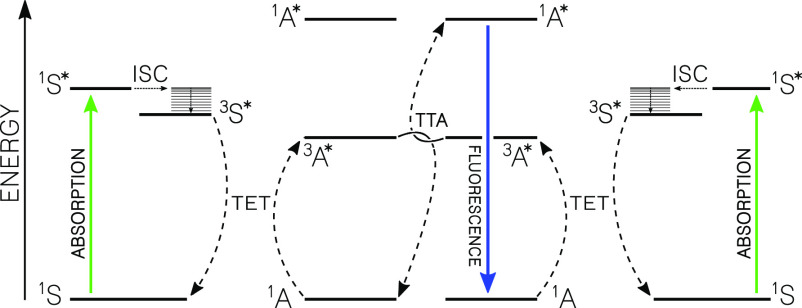
Jablonski diagram depicting the energy levels and transfer steps involved in photon upconversion by triplet–triplet annihilation (TTA-UC). S = sensitizer; A = annihilator.

The ability to manipulate incoming sunlight to improve the spectral matching for different applications has seen great progress in recent years. Shockley and Queisser famously derived the intrinsic limit for p–n junction solar cell efficiencies more than 50 years ago,^1^ and two different ways of breaking this limit have gained extensive attention. To mitigate thermalization losses due to excess energy in the photons arriving at the junction, singlet fission (SF), a process in which a singlet excited state may be converted into two triplet excited states, has been proposed.^2^ The reverse of SF, that is, combining two low-energy photons into one high-energy excited state (exciton), is referred to as photon upconversion.^3,4^ This could be used to manage the losses associated with insufficient photon energy, thus expanding the spectral range of the solar cell.^5,6^ The process may operate by several different mechanisms, and triplet–triplet annihilation upconversion (TTA-UC) is one possible mechanism (Figure 1) that may proceed utilizing low-intensity, noncoherent light. Thus, TTA-UC is of special interest not only for photovoltaics^7−10^ but also in other solar energy conversion applications, such as photocatalysis^11−13^ and photochemistry.^14−16^ Two compounds are needed in this process: a sensitizer (S) and an annihilator (A). The sensitizer molecule absorbs one long wavelength photon and undergoes rapid intersystem crossing (ISC). Dexter-type triplet energy transfer (TET)^17^ from the sensitizer subsequently populates the first triplet excited state (^3^A*) of the annihilator molecule. This is then followed by TTA between two triplet excited annihilators, forming one ground-state (^1^A) and one singlet excited-state (^1^A*) annihilator, of which the latter may emit one shorter-wavelength photon.

The most efficient systems developed to date are liquid solutions of S and A,^18^ allowing for swift diffusion of long-lived triplets and thus promoting the short-range Dexter-type events. For practical applications solid-state solutions are however needed, introducing obstacles in terms of inefficient energy transfer due to spatial separation between the molecules.^19^ One way to mitigate these obstructions is by allowing the TTA event to proceed in an intramolecular fashion in addition to the intermolecular counterpart, which would require excitons to diffuse within the annihilator instead of relying on molecular diffusion.^20−33^ Our group has previously studied dendrimeric and oligomeric frameworks of covalently bonded 9,10-diphenylanthracene (DPA) units, showing that intramolecular TTA-UC in rigid environments is promoted by large annihilator frameworks.^23^ This behavior was further investigated using porphyrin–anthracene complexes, showing that parasitic back energy transfer from the anthracene unit to the covalently attached porphyrin is subdued as the anthracene unit increases in size.^24,32^ Several recent contributions have investigated different types of dimeric annihilators based on DPA^29,31^ and tetracene,^22,33^ respectively, in which intramolecular TTA (intra-TTA) is hypothesized to occur. A full mechanistic picture of intramolecular upconversion is however lacking, and different models for the energy transfer events have been proposed.^23,29,31,33^

In this study, four novel dimeric compounds based on DPA have been synthesized and investigated. The dimeric nature allows these annihilators to hold two triplets (triplet excited states) simultaneously, thus enabling intra-TTA in addition to conventional intermolecular TTA. The importance of structure and molecular geometry has been thoroughly investigated for SF and has been shown to play an important role in governing the SF efficiency and rate.^34−40^ In contrast, the structural effects of intra-TTA have not been fully understood. These dimers allow us not only to investigate the mechanism of intramolecular upconversion but also to draw conclusions with regard to how structural motifs of the annihilators relate to the upconversion performance.

## Results and Discussion

### Design and Synthesis of DPA Dimers

Synthesis details (Figure S1) as well as the proton and carbon NMR spectra for the dimers (Figures S2–S9) are found in the Supporting Information. The dimers consist of two anthracene units which are connected in four different ways. Three of the dimers, 1,2-bis(10-phenylanthracen-9-yl)benzene (1,2-DPA_2_), 1,3-bis(10-phenylanthracen-9-yl)benzene (1,3-DPA_2_), and 1,4-bis(10- phenylanthracen-9-yl)benzene (1,4-DPA_2_), have a central phenyl ring, and the numeric indices indicate at which positions the anthracenes connect to the ring. In comparison, 10,10′-diphenyl-9,9′-bianthracene (9,9′-PA_2_) lack the central phenyl ring connector, and instead the anthracene moieties are directly connected at their respective 9-positions.

### Photophysical Characterization

**Figure 2:**
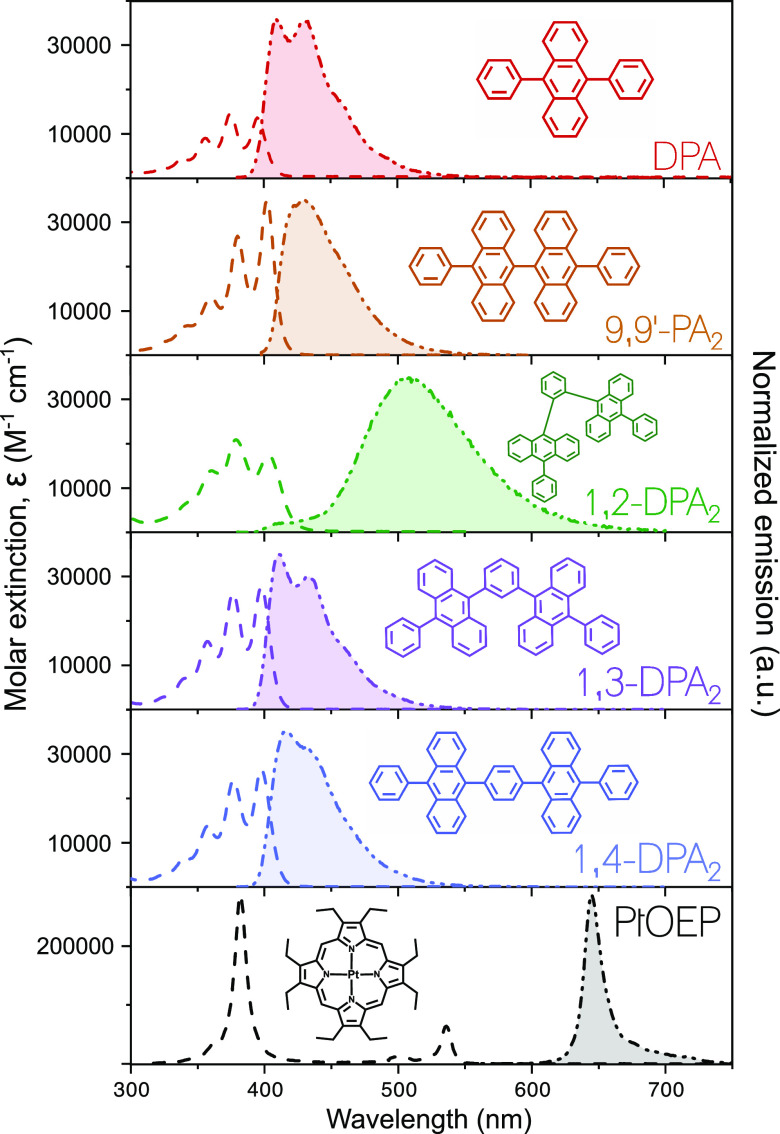
Absorption (dashed) and emission (dot–dash) spectra of the investigated annihilators and the sensitizer PtOEP.

Figure 2 presents the absorption and fluorescence spectra of DPA and the dimer compounds, alongside that of platinum octaethylporphyrin (PtOEP). The latter was employed as the sensitizer during TTA-UC owing to its suitable energetics, strong absorption, and rapid intersystem crossing.^41^ The molar absorptivity of the dimers is roughly two times higher than for DPA, which is expected given the dimeric nature of these compounds. The vibronic progression is clearly visible in the absorption features of all dimers, with relative intensities of the vibronic peaks being the major difference between different spectra. Only minor shifts of the peak positions were observed. More pronounced differences were found upon investigation of the fluorescence properties. All compounds have a high fluorescence quantum yield close to unity in deaerated toluene, thus fulfilling one important prerequisite to perform well as an annihilator in TTA-UC.^4^ The fluorescence lifetimes are slightly shorter than for DPA for all dimers except 1,2-DPA_2_. Although not quantitatively reflected in the increased molar absorptivities, the faster radiative rates of the dimers show that the linked DPA chromophores interact electronically. Too weak emission at 410 nm from 1,2-DPA_2_ at room temperature hindered us from properly monitoring the lifetime of monomeric fluorescence, i.e., fluorescence originating from the isolated, noninteracting DPA moieties. The tabulated lifetime at 410 nm was instead measured at 93 K where short-wavelength fluorescence dominates. The findings are summarized in Table 1. A substantial red shift is seen for the fluorescence of 1,2- DPA_2_, with only a small fraction of the fluorescence taking place at shorter wavelengths at room temperature (Figure S11). This behavior is ascribed to the formation of an excited dimer, or *excimer*, upon excitation and has previously been observed for a number of different annihilator molecules.^42−44^ The creation of excimers is normally highly dependent on chromophore concentration as it is formed through interactions between a ground-state and an excited-state molecule. The fluorescence from 1,2-DPA_2_ does not show this dependence on concentration. The long-wavelength emission is dominating also for low-concentration samples, and thus, the excimer is believed to form by means of intramolecular interactions.^42,45,46^ The behavior and kinetics of 1,2-DPA_2_ were investigated thoroughly but are not the main focus of this paper, and the interested reader is referred to Section 3 of the Supporting Information. It is important to point out that, at room temperature, the lifetime of the initially excited singlet state of 1,2-DPA_2_ is very short (<10 ps) due to quantitative transformation into the singlet excited excimer, which is why all emission emanates from the excimer irrespectively if populated through TTA or direct excitation. A large fraction of this emission lies at longer wavelength than 532 nm and is, thus, not strictly speaking upconverted.

**Table 1:** Photophysical Properties of DPA and Dimers

	*E*_0–0_ (nm)^a^	λ_em_ (nm)^b^	ϕ_f_	τ_f_ (ns)^c^
DPA	393 (13.6)	409	1.00	6.91
9,9′-PA_2_	402 (35.0)	431	1.00	6.56
1,2-DPA_2_	403 (17.3)	511	0.95	7.32^d^, 44.0^e^
1,3-DPA_2_	398 (27.7)	411	0.96	5.85
1,4-DPA_2_	398 (26.7)	416	0.95	4.56

a Wavelength of 0 → 0 transition and molar absorptivity, ε (×10^3^ M^–1^ cm^–1^).

b Wavelength of maximum emission upon 377 nm excitation at 295 K.

c Fluorescence lifetime probed at 410 nm and 295 K.

d Probed at 410 nm and 93 K.

e Probed at 510 nm and 295 K.

### Upconversion Study

The upconversion potential of the four dimers has been evaluated in a series of experiments. The upconverting samples consisted of 6.6 μM PtOEP and 1 mM (DPA) or 0.5 mM (dimers) annihilator, which upon 532 nm excitation produced bright upconverted fluorescence (Figures S16–S17). No upconverted emission could be detected when PtOEP was absent from the samples.

A figure of merit which is of big importance when evaluating upconverting systems is the upconversion quantum yield, ϕ_UC_. It is defined as the number of emitted high-energy photons compared with the number of absorbed low-energy photons and can thus take a maximum value of 50%.^47^ The efficiency is dependent on the quantum yield of all steps leading up to the emission of photons in accordance with eq 1:
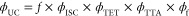
1

Here, ϕ_ISC_ is the intersystem crossing quantum yield of the sensitizer, ϕ_TET_ is the quantum yield of triplet energy transfer from sensitizer to annihilator, ϕ_TTA_ is the quantum yield of triplet–triplet annihilation, and ϕ_f_ is the fluorescence quantum yield of the annihilator. The spin statistical factor, *f*, defines the fraction of excited annihilator triplets that go on to create an emissive singlet excited state following TTA.^18^ Evaluating each of the terms in eq 1 is not viable, and instead the method of relative actinometry (eq 2) is used:
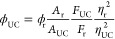
2Here, ϕ_r_ is the known fluorescence quantum yield of a reference compound, *A*_*i*_ is the absorption at the excitation wavelength, *F*_*i*_ is the integrated emission intensity, and η_*i*_ is the refractive index. Subscripts r and UC denote reference sample and upconversion sample, respectively. Rhodamine 6G in air-saturated ethanol was employed as the reference compound (ϕ_f_ = 0.95)^48^ during UC measurements.

All dimers exhibit efficient upconversion upon 532 nm excitation (Table 2). 9,9′-PA_2_, 1,2-DPA_2_, and 1,4-DPA_2_ perform similarly, yielding a quantum yield of around 15%. Interestingly, 1,3-DPA_2_ outperforms the other dimers, showing an impressive 21.2% ϕ_UC_ which is comparable with 24.0% for DPA. Dimerization in the meta-position has previously yielded higher UC efficiencies than the corresponding para- and ortho-connected annihilators,^31^ and our results indicate that connections in the meta-position are beneficial at least for DPA-type dimer annihilators. The calculated thermodynamic driving forces for TTA, i.e., 2 × *E*(T_1_) – *E*(S_1_), are similar for all annihilators and do not explain the differences in our results.

**Table 2:** Experimentally and Computationally Determined Parameters Important to the Upconversion Process

	Φ_UC_^a^	*I*_th_^b^ (mW/cm^2^)	*k*_TET_^c^ (×10^9^ M^–1^ s^–1^)	*k*_TTA_^d^ (×10^9^ M^–1^ s^–1^)	τ_T_^e^ (ms)	*k*_T_^f^ (×10^3^ s^–1^)	*E*(S_1_)^g^ (eV)	*E*(T_1_)^h^ (eV)	2 × *E*(T_1_) – *E*(S_1_)^j^ (eV)
DPA	0.240	15	1.78	3.01	5.5	0.18	3.05/3.15	1.72^i^	0.29
9,9′-PA_2_	0.150	605	0.99	3.73	0.56	1.79	2.86/3.08	1.72	0.36
1,2-DPA_2_	0.140	142	0.95	2.89	0.80	1.25	2.85/3.08	1.71	0.34
1,3-DPA_2_	0.212	44	1.04	2.81	4.7	0.21	3.04/3.12	1.72	0.32
1,4-DPA_2_	0.149	1343	0.96	4.00	0.29	3.44	3.06/3.12	1.72	0.32

a Upconversion quantum yield relative to a theoretical maximum of 0.5 (50%).

b Threshold intensity. Individual values have been normalized with respect to slight deviations in [S] between samples.

c Rate constant for triplet energy transfer from PtOEP.

d Rate constant for triplet–triplet annihilation.

e First triplet excited-state lifetimes.

f Rate constant for intrinsic triplet decay.

g Energy of the first singlet excited state as calculated with TD-DFT (B3LYP/6-31G**)/calculated from the 0 → 0 transition of the absorption spectra. See the Supporting Information for calculation details.

h Energy of the first triplet excited state as calculated with TD-DFT.

i Experimental literature value^50^ is 1.77 eV.

j Thermodynamic driving force for TTA.

In order to understand why 1,3-DPA_2_ exhibits a higher ϕ_UC_ than the other dimers, further experiments aimed at understanding the kinetics involved were performed. Efficient triplet energy transfer between sensitizer and annihilator is a feature present in all high-performing UC systems and may be investigated using Stern–Volmer quenching (eq 3):
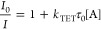
3

**Figure 3:**
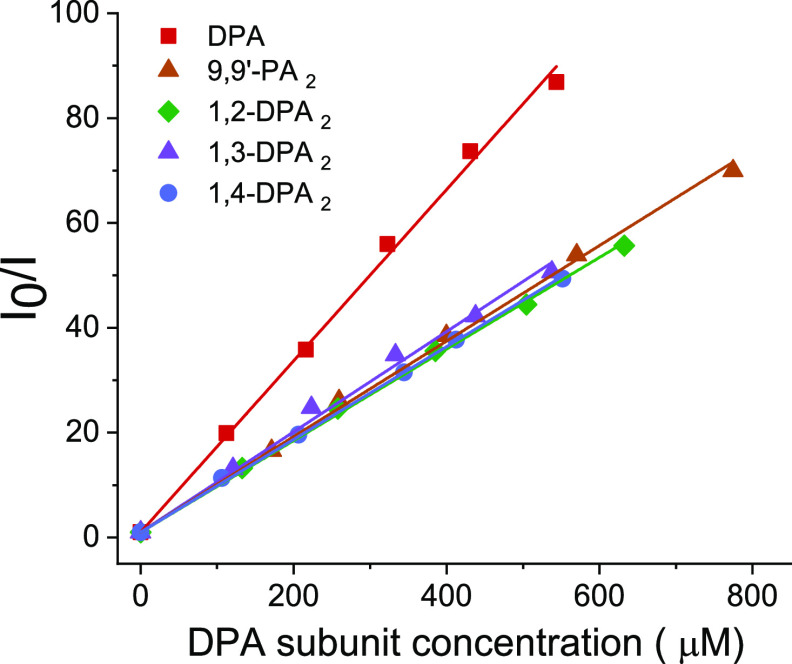
Stern–Volmer plots of TET from PtOEP (3.4 μM) to the five annihilator compounds.

*I* and *I*_0_ are the quenched and unquenched donor emission, respectively, *k*_TET_ is the rate constant for TET from donor to acceptor, τ_0_ is the lifetime of the unquenched donor, and [A] is the quencher concentration. The quenching of PtOEP with the five different annihilators was determined by monitoring the phosphorescence of PtOEP, and the resulting Stern–Volmer plots are presented in Figure 3. The unquenched lifetime of PtOEP was determined to be 95 μs in deaerated toluene (Figure S20), and TET from PtOEP to the annihilators is found to be very efficient in all cases. The *k*_TET_ rate constants of the dimers are roughly half that of DPA (measured in DPA subunit concentrations, Table 2), which is reasonable given that the lower diffusivity of the dimers is compensated for by a larger collision radius according to the Smoluchowski equation.^49^ All TET efficiencies are 99% or higher for DPA subunit concentrations of [A] = 1 mM, and it was concluded that the TET step is not the determining factor when evaluating these dimers against each other.

Another important figure of merit when evaluating upconverting systems is the threshold intensity, *I*_th_. This is the excitation intensity at which ϕ_UC_ reaches 50% of a systems’ maximum and is related to system parameters as in eq 4:
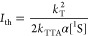
4

**Figure 4:**
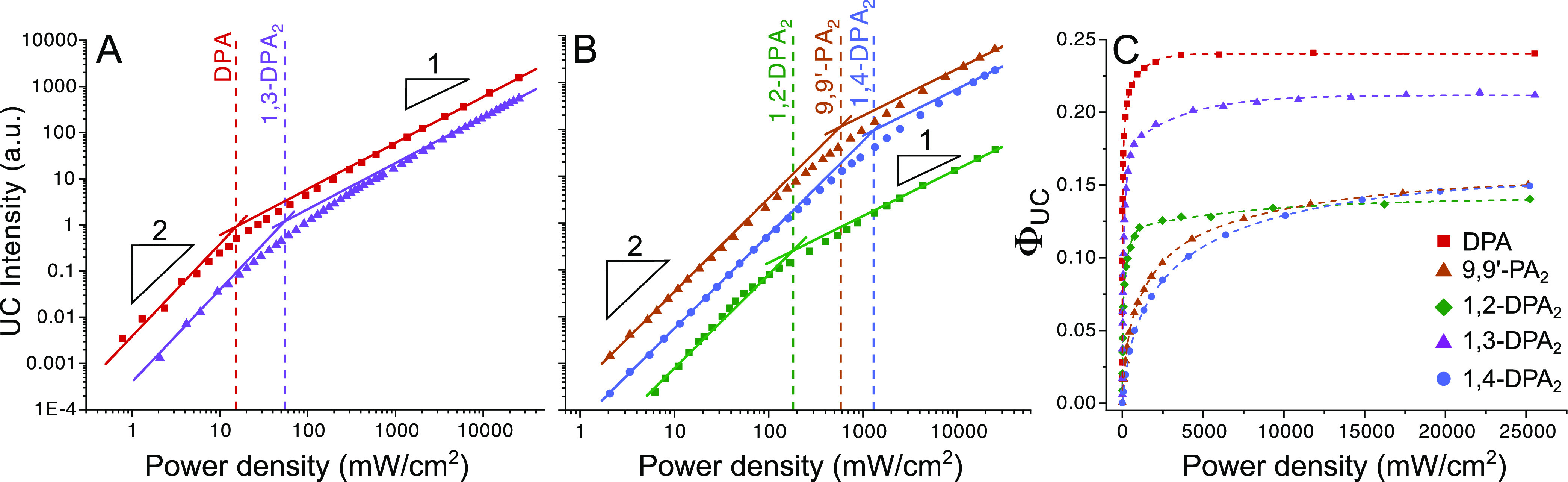
(A, B) Double-logarithmic plots of upconversion emission intensity versus excitation power density (532 nm). The threshold intensity *I*_th_ for each annihilator is indicated by vertical dashed lines, and quadratic and linear slopes (solid lines) for each compound are included for facile evaluation of *I*_th_. Individual plots have been vertically shifted for clarity purposes. (C) UC quantum yield versus excitation power density for the annihilators.

The absorption cross section, α, and ground-state concentration, [^1^S], of the sensitizer are the same in all upconverting samples. Only the rate of intrinsic triplet decay of the annihilator, *k*_T_, and the rate of TTA, *k*_TTA_, will thus influence differences in *I*_th_ between annihilators. The *I*_th_ value defines the crossing point between a quadratic region where the supply of annihilator triplets is the limiting factor and a linear region where UC proceeds with the highest possible efficiency. For practical applications it is desirable to have as low an *I*_th_ as possible, as this facilitates possible applications using solar energy (the power density from the sun between 470 and 550 nm, i.e., the absorption range for the PtOEP Q-band, is approximately 15 mW/cm^2^ under AM1.5 solar irradiation). The excitation power density dependence on the UC emission intensity of our systems is presented in Figure 4. 1,2-DPA_2_ and 1,3-DPA_2_ have *I*_th_ values reasonably close to what is usually seen for compounds based on DPA (142 and 44 mW/cm^2^, respectively) but are significantly higher than the acquired value for DPA (15 mW/cm^2^, see also Table 2). The less congested dimers 9,9′-PA_2_ and 1,4-DPA_2_ have *I*_th_ values of 605 and 1343 mW/cm^2^, respectively, which is more than 1 order of magnitude higher than that of DPA. At closer examination of Figure 4B it becomes obvious that the linear regime (slope 1) has not been fully reached by either 9,9′-PA_2_ or 1,4-DPA_2_ at our setups maximum power density, and the presented values thus represent a lower estimate of their respective *I*_th_ value. The spread in *I*_th_ values is a bit surprising given that other studies on DPA-based molecules have shown a narrow distribution of threshold intensities and often close to that of DPA.^29,31,51^ These unexpected results were further analyzed by examining eq 4 and seeking to determine the parameters involved.

**Figure 5:**
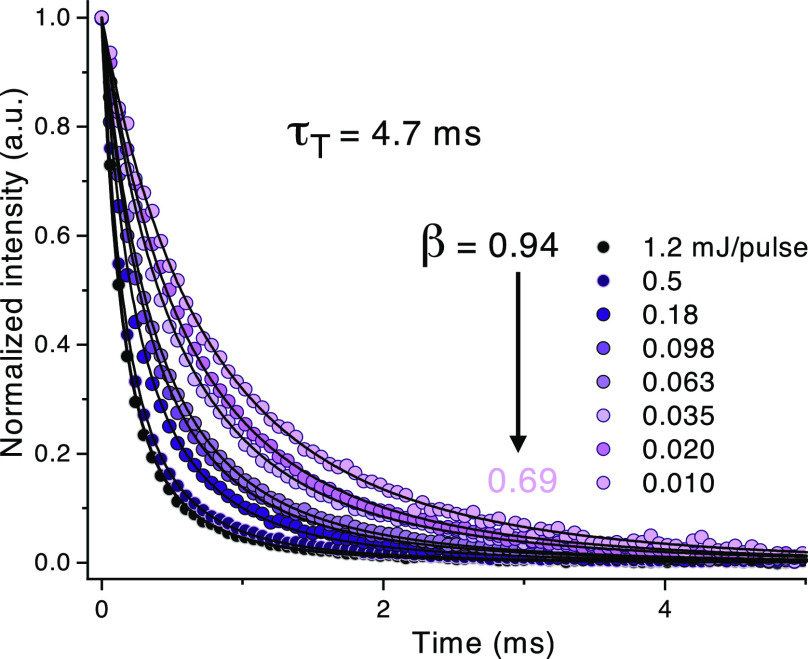
Time-resolved, delayed upconverted fluorescence from 1,3-DPA_2_ in the presence of PtOEP. The emission is measured at 430 nm and at different pump excitation intensities (532 nm). Solid lines are best global fits to eq 5.

Vital to an efficient upconversion process are long-lived annihilator triplet states, as this allows for diffusion-mediated annihilation to proceed more efficiently. Using time-resolved emission measurements and a fitting procedure based on eq 5, the annihilator triplet lifetimes, τ_T_, and related *k*_T_ rate constants could be determined (Table 2). The annihilator triplet state may decay by both first- and second-order channels, and the observed kinetics of the upconverted fluorescence will thus depend on the annihilator triplet concentration:^52^
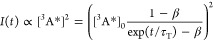
5Here, *I*(*t*) is the emission intensity, β is a dimensionless parameter between 0 and 1 expressing what fraction of initial decay is governed by second-order channels (with β = 1 meaning all initial decay is of second order), and *t* is time. Full details are found in the Supporting Information. As was the case with *I*_th_, these compounds show a rather broad distribution of triplet lifetimes, ranging from 291 μs for 1,4-DPA_2_ to 5.5 ms for DPA (Figures S21–S24 and Table 2). 1,3-DPA_2_ (Figure 5) has the most long-lived triplet out of the dimers, which explains the high ϕ_UC_ as well as the relatively low *I*_th_, while the shorter triplet lifetimes of 9,9′-PA_2_ and 1,4-DPA_2_ are consistent with higher *I*_th_ values. The longer lifetime of the triplet state in 1,3-DPA_2_ could potentially be explained by the decreased electronic coupling between the moieties due to linkage in the meta-position,^31,53−55^ thus better retaining the properties of the DPA monomer. This notion was strengthened by calculations of the electronic coupling between the triplet excited states of the dimers (see Supporting Information, Section 4.4). The second parameter that affects the threshold intensity is the rate of annihilation. *k*_TTA_ was determined for all compounds using nanosecond transient absorption, monitoring UC samples at 430 nm and at 646 nm following 532 nm excitation. The transient signals are presented in Figures S26–S30, and a global fitting procedure was used to obtain the rate constants of interest (the full procedure is explained in the Supporting Information, Section 4.3). The *k*_TTA_ rates are quite similar (see Table 2), but interestingly 9,9′-PA_2_ and 1,4-DPA_2_ show slightly elevated rates, even compared to DPA.

### Intramolecular Upconversion in Solution

The design of our annihilators was in part based on the possibility of populating the molecules with two triplets simultaneously, as this enables intramolecular upconversion (hereon after referred to as intra-UC). Instead of relying on molecular diffusion, this mechanism may rely on the diffusion of excitons within a molecular system. Several recent studies exploiting dimeric,^22,29,31,33^ polymeric,^20,21,26−28,30^ and oligomeric^23^ structures have sought to investigate the influence of this additional pathway. In a recent study, Gao et al. studied structurally similar DPA derivatives which were separated with additional phenyl groups.^31^ The coupling pattern, para-, ortho-, meta-, was however the same as in the present study. Interestingly, the ϕ_UC_ trend follows what we observe here: DPA > meta- > ortho- and para-. However, in their case no significant difference in the triplet lifetime was observed between the derivatives and can thus not explain this trend. The difference was instead assigned to greater intra-TTA contributions for the meta-coupled dimer, which were indicated by means of magnetic-field-dependent measurements of the UC performance. It should be noted that their reported triplet lifetime of DPA was roughly five times shorter than that reported herein. This discrepancy is likely caused by differences in the type of measurement and fitting procedures, as Gao et al. used transient absorption without taking second-order channels into account during fitting. Figure S25 highlights that a single-exponential tail fit significantly underestimates the triplet lifetime, especially in systems with high TTA efficiencies.

**Scheme 1:**
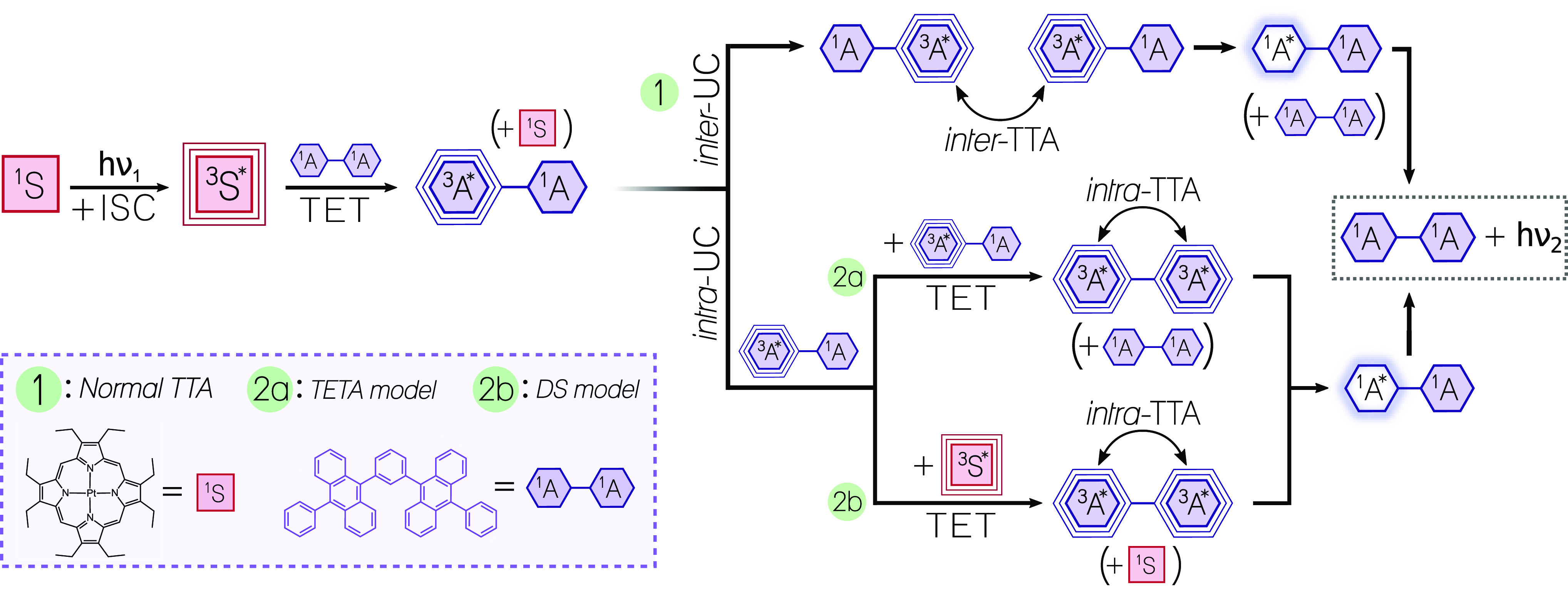
Intermolecular Upconversion vs Two Suggested Models for Intramolecular Upconversion: TETA and DS Schematic of suggested pathways for triplet–triplet annihilation upconversion with dimeric annihilator compounds (here represented by 1,3-DPA_2_). Designations: S = sensitizer, A = annihilator moiety (spin multiplicity denoted by left superscript), * = excited state. Upon light absorption (*h*ν_1_) and rapid intersystem crossing (ISC), the sensitizer populates the triplet excited state of one annihilator moiety through triplet energy transfer (TET). The TTA event forms a singlet excited state, and one high-energy photon (*h*ν_2_, ν_2_ > ν_1_) is emitted. (1) Conventional intermolecular TTA between two triplet excited dimers. (2a) TET between annihilators (TETA) model: The triplet excited dimer becomes doubly excited following TET from another triplet excited annihilator. (2b) Double sensitization (DS) model: The ground-state moiety of the singly triplet excited dimer is populated with another triplet following TET from a sensitizer molecule.

In this section, the quest to fully understand intra-UC is continued, as there are still disagreements on how the actual mechanism of intra-UC proceeds in solution-based systems. Two different mechanisms are predominantly considered in the literature, and these are schematically presented in Scheme 1 alongside the intermolecular upconversion (inter-UC) route (pathway 1 in Scheme 1) always present in solution-based systems. The *triplet energy transfer between annihilators* model (TETA model, pathway 2a in Scheme 1) has been suggested by several authors^29,31^ and is based on the interactions between two multichromophoric triplet excited annihilators, ^3^A*–^1^A (in this section, annihilators are assumed to have the capacity of holding two triplets simultaneously and are then designated ^3^A*–^3^A*). Normally the interaction between two ^3^A* leads to an intermolecular TTA event where the *f* factor (see eq 1) determines the yield of singlet excited states. However, when two ^3^A*–^1^A interact there is the additional possibility of a triplet energy transfer step from the triplet moiety of one annihilator to the ground-state moiety of the other, creating one ^3^A*–^3^A* and one ^1^A–^1^A annihilator. An intra-TTA event between two triplets then rapidly gives the singlet excited state ^1^A*–^1^A and subsequent emission of an upconverted photon. It has been suggested that the TET step between two singly excited dimeric annihilators proceeds statistically, meaning that half of these interactions lead to ^3^A*–^3^A*, while only 25% proceeds through the common intermolecular TTA pathway to produce the singlet excited state ^1^A*–^1^A.^31^

The *double-sensitization model* (DS model, pathway 2b in Scheme 1) relies not on TET between annihilators but rather on a second TET step from a triplet excited sensitizer (^3^S*) to the ground-state moiety of ^3^A*–^1^A, ultimately leading to a ^3^A*–^3^A* annihilator which can proceed to perform intra-TTA. This model has been employed in a multitude of other studies,^22,23,28,33^ but the conclusions of different investigations in low-viscosity media vary significantly. In pursuit of elucidating the nature of intra-UC, steady-state and time-resolved simulations of the presented models were performed. The experimentally determined parameters for each annihilator (i.e., *k*_TET_, *k*_T_, and *k*_TTA_) were used during simulations, and the detailed description of the kinetic model is presented in the Supporting Information.

**Figure 6:**
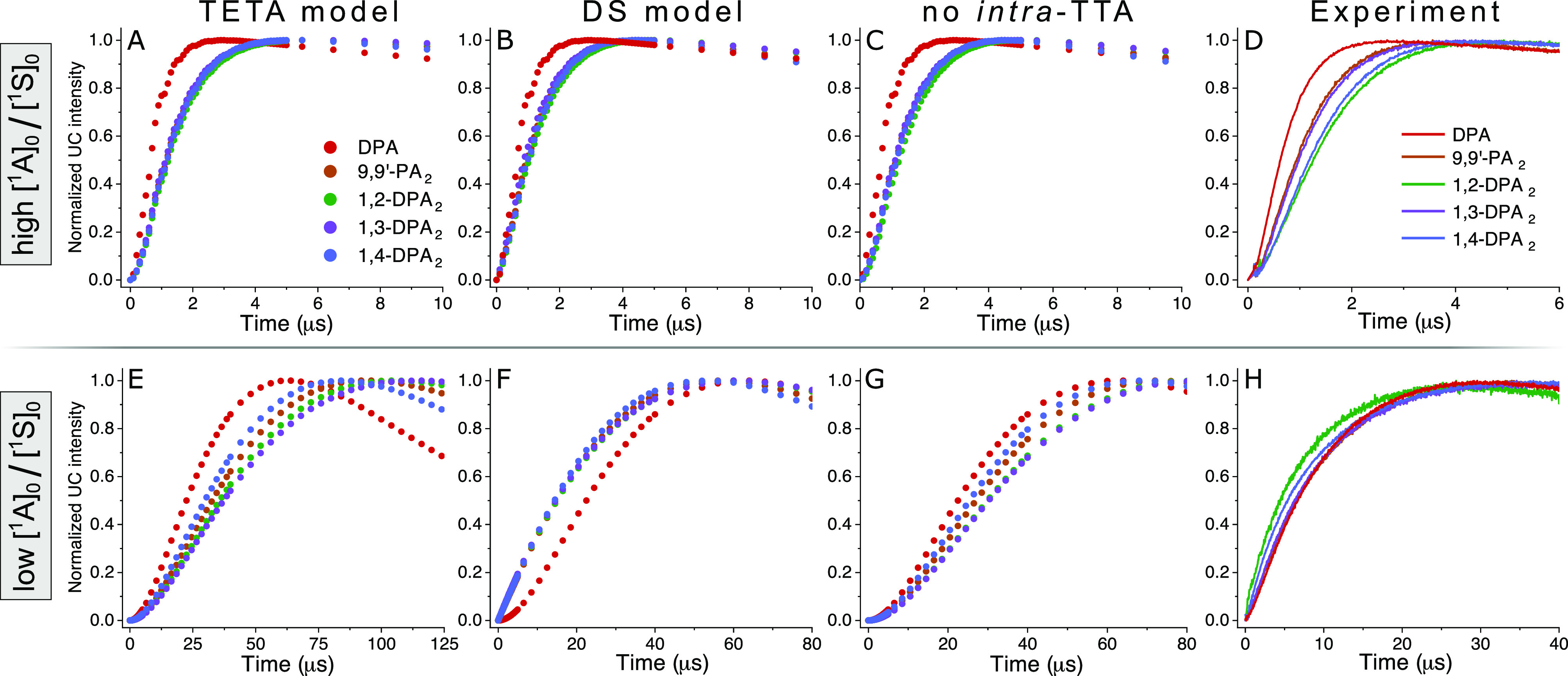
Simulation results for the (A, E) TETA model, (B, F) DS model, (C, G) model where intra-TTA contributions are disallowed, and (D, H) experimental kinetic traces from delayed UC fluorescence emanating from (A–D) samples with [^1^S]_0_ = 5 μM and [^1^A]_0_ = 1 mM (i.e., [^1^A]_0_/[^1^S]_0_ = 100), and (E–H) samples with [^1^S]_0_ = 100 μM and [^1^A]_0_ = 5 μM (i.e., [^1^A]_0_/[^1^S]_0_ = 0.05). Emission monitored at 430 or 510 nm (for 1,2-DPA_2_).

The two models have been evaluated for two different sample conditions: typical UC conditions (i.e., [^1^A]_0_ = 1 mM, [^1^S]_0_ = 5 μM), which will be referred to as high annihilator to sensitizer ratios ([^1^A]_0_/[^1^S]_0_), and conditions with low [^1^A]_0_/[^1^S]_0_ ratios (i.e., [^1^A]_0_ ≈ 10 μM, [^1^S]_0_ = 100 μM). This has been done in order to investigate the expected influence from each intra-UC mechanism separately and to draw conclusions on in which regimes each model is valid. Time-resolved simulations were performed, which are presented in Figure 6. At high [^1^A]_0_/[^1^S]_0_ ratios, the TETA model predicts that the dimer UC emission kinetics are slightly slower than in the DS model. However, both models predict that the kinetics get slower upon lowering [^1^A]_0_. The largest difference between the models is the evolution of early time kinetics, which are related to the TET events. No major difference between the models is discernible at high [^1^A]_0_/[^1^S]_0_ ratios (Figure 6A, B), but upon going to low [^1^A]_0_/[^1^S]_0_ it is clear that the DS model predicts that dimers will have a much faster rise time relative to that of DPA, a behavior not predicted by the TETA model (Figure 6E, F). Given the nature of the DS model, the faster early time kinetics would be expected as the intra-UC pathway depends on TET from ^3^S*, an event most likely to take place early on.

To further elucidate the differences between the two models, steady-state simulations were also performed. Figure S33 shows the expected steady-state delayed UC fluorescence following 532 nm continuous-wave (cw) excitation for high [^1^A]_0_/[^1^S]_0_ ratios, with [^1^A]_0_ ranging from 0 to 5 mM. It is clear that the potential contribution from intra-UC is indiscernible at higher [^1^A]_0_ according to the DS model, which is expected as generated ^3^S* will quickly transfer their energy to surrounding ground-state ^1^A, thus swiftly depleting the ^3^S* population and hindering subsequent TET events needed to produce the doubly excited dimers (Figure S34). On the contrary, intra-UC contributions are expected to increase with [^1^A]_0_ in the TETA model, as this pathway ultimately depends mainly on [^3^A*–^1^A] (Figure S33B). Conversely, increasing [^1^S]_0_ to 100 μM gives rise to interesting behavior at lower [^1^A]_0_ (<10 μM), with the DS model predicting that intra-UC will dominate, allowing annihilator dimers to exhibit stronger UC fluorescence than their corresponding monomer under such conditions (Figure S35A). The TETA model predicts the resulting UC intensity to be lower than that of DPA also under these conditions (Figure S35B).

Samples of low and high [^1^A]_0_/[^1^S]_0_ ratios were prepared, and the behavior observed experimentally was compared with that predicted by each model. Time-resolved measurements of the UC emission display a marked difference in the kinetics when going from high (Figure 6D) to low (Figure 6H) [^1^A]_0_/[^1^S]_0_. It is clearly seen that the rise time of the UC emission of the dimers at low [^1^A]_0_/[^1^S]_0_ is faster relative to that of DPA (red line), even to such an extent that some dimers develop the UC emission faster than DPA. The evolution of the rise time kinetics in the dimers is compatible with the DS model only, which indeed predicts that the dimer UC emission will develop faster than that of DPA at low [^1^A]_0_/[^1^S]_0_ (Figure 6F). Interestingly, the kinetic evolution of the individual dimers differs slightly as well, with 1,2-DPA_2_ in particular showing a substantial shortening of its rise time compared with the other annihilators, indicating a stronger influence from intra-UC. While this could potentially be ascribed to differences in the intra-TTA event, simulations firmly establish that the observed differences are in fact determined by the second sensitization step (Figure S37). We find no reason to believe that the ortho-coupling in 1,2-DPA_2_ would cause a more efficient second sensitization step and ascribe the observed differences between individual dimers to experimental uncertainty.

**Figure 7:**
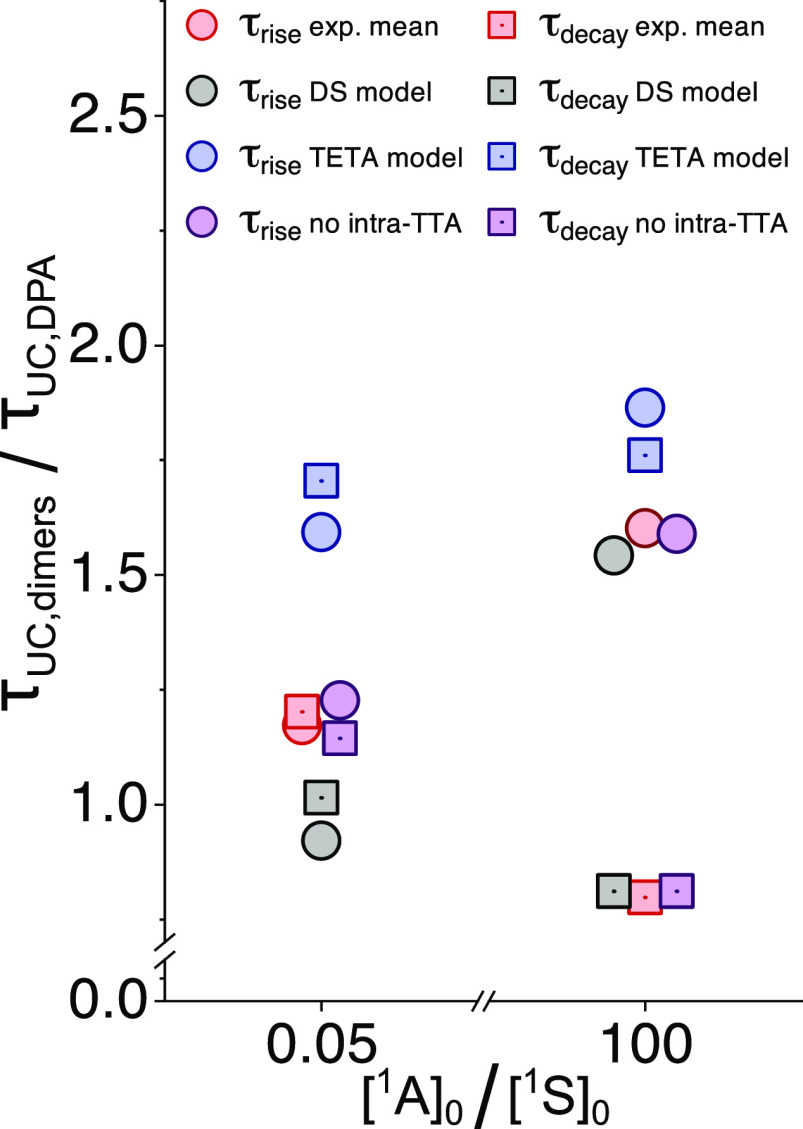
Comparison of relevant time constants. The mean value for the dimeric annihilators is compared to the value of DPA at [^1^A]_0_/[^1^S]_0_ = 0.05 or 100. The rise times (τ_rise_) and decay times (τ_decay_) are evaluated at the peak and 1/*e* values of the normalized emission traces, respectively.

To quantify our analysis, the mean value of the rise times and decay times of our dimers was compared with that of DPA (Figure 7). The experimental ratios (red symbols) were then compared to the ratios predicted by the DS model (black symbols) and the TETA model (blue symbols) and for a scenario where intra-UC is disallowed (purple symbols). This was made at the two previously used [^1^A]_0_/[^1^S]_0_ ratios, and the results show a striking agreement between our experimental data and the model where no intra-UC occurs. The experimental data also show good agreement with the DS model, while the accordance to the TETA model is rather poor. Even though our quantified time-resolved data may be explained without involving the intra-UC pathway, such a description is not sufficient to explain the appearance of the kinetics in Figure 6H. Specifically, the immediate emergence of UC emission from the dimers during the first tens of microseconds is most likely a result of the DS mechanism and is particularly evident in the traces of 1,2-DPA_2_ (green) and 1,4-DPA_2_ (blue, see Figure 6F and 6G for a comparison of the DS model kinetics and the kinetics expected if no intra-UC contributions are present). It should be noted that the remarkable agreement between experimental data and the DS model at high [^1^A]_0_/[^1^S]_0_ ratios (Figure 7) is caused by the fact that no intra-UC is expected under such conditions, and the data is thus expected to coincide with that of the model where intra-UC is disallowed completely.

Figure S38 shows the results from the steady-state measurements, where [^1^A]_0_ has been varied while keeping [^1^S]_0_ at 100 μM throughout, except for the rightmost data point which is given for [^1^A]_0_ = 1 mM, [^1^S]_0_ = 6.6 μM. No clear-cut interpretation is readily obtained from these data, with the only prominent feature being the relative 1,4-DPA_2_ emission increasing as the [^1^A]_0_/[^1^S]_0_ ratio gets lower. This could indicate that there are some contributions from intra-TTA in 1,4-DPA_2_ (given that the DS mechanism is active). However, 9,9′-PA_2_ and 1,3-DPA_2_ do also show signs of increased relative emission, although not as systematically as 1,4-DPA_2_, while 1,2-DPA_2_ exhibits similar behavior independent of [^1^A]_0_. The steady-state measurements are rather sensitive to experimental errors, with the evaluation of precise intensities being subject to substantial inner-filter effects, high sensitivity to exact sample concentrations, and possible oxygen contamination. Similar measurements on tetracene dimers by Pun et al. have however proved useful previously, specifically in contexts where low k_TTA_ rates are measured for the upconverting materials.^33^ In this case the impact of the DS mechanism is manifested also at relatively high [^1^A]_0_/[^1^S]_0_ ratios, thus facilitating comparisons between annihilators where intra-TTA is allowed and forbidden, respectively.

**Figure 8:**
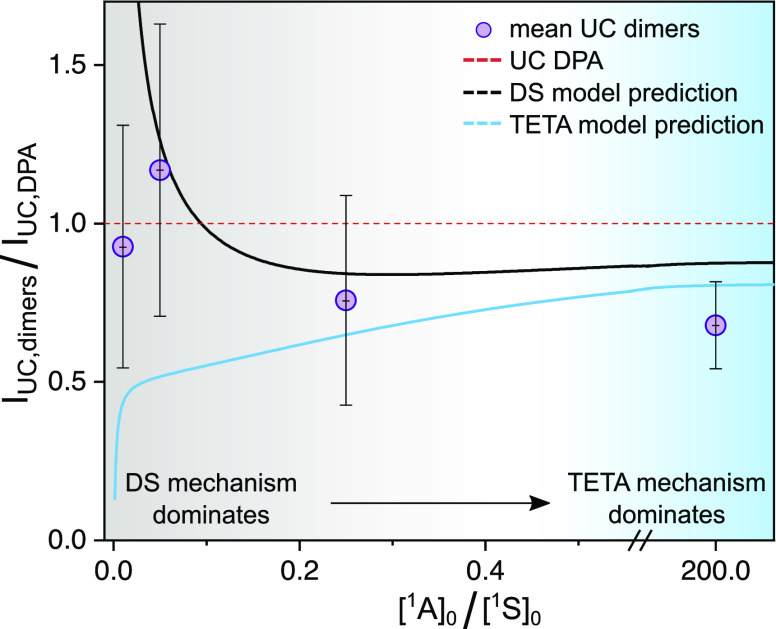
Comparison between the UC emission of DPA and dimers as a group. The abscissa signifies the ratio between ground-state annihilator and sensitizer concentrations, and the ordinate the mean relative UC emission of the dimers compared to that of DPA. Purple circles are the experimental mean values of dimer UC emission divided by that of DPA and are compared with the corresponding intensities predicted by the DS (black solid line) and TETA (blue solid line) models. The red dotted line represents the UC emission of DPA and is included as a reference point.

Despite the fact that our experiments did not yield any obvious signatures indicating the presence of the TETA mechanism, a closer examination of our steady-state data might shine some additional light on this matter. Although the data presented in Figure S38 show differences between the performances of individual dimers, our time-resolved measurements indicate that all investigated dimeric annihilators perform intra-TTA to some extent. It can therefore be helpful to look closer at the dimers as a group to understand the role of the intra-UC mechanism. Comparing the experimental mean performance of the dimers to the two proposed models then indicates which model explains the UC performance of the dimers as a group. We have previously established that the annihilator triplet lifetime is a crucial parameter which dictates much of the UC performance, but it cannot on its own explain the observed differences in UC efficiencies. In fact, the DS and TETA models predict slightly different UC efficiencies at different conditions, with the former complying better with our experimental findings at low [^1^A]_0_/[^1^S]_0_ ratios but the latter agreeing more with experiments at high [^1^A]_0_/[^1^S]_0_ ratios (Figure 8). Even though this transition from conditions under which the DS mechanism dominates to one where TETA dominates is not unambiguous, this change is expected given that the intra-UC mechanism in the DS and TETA models depends on [^3^S*] and [^3^A*-^1^A], respectively, which are high at each extreme of Figure 8. The correspondence between simulation and experiment is however not without fault, and the relatively low UC efficiencies of the dimers compared to DPA at high [^1^A]_0_/[^1^S]_0_ ratios (rightmost point in Figure 8) could possibly be due to differences in the intramolecular spin-statistical factor. This factor has not been included in our models but has been suggested to be rather low in compounds similar to ours.^31^ Moreover, the TETA mechanism which dictates intra-UC at high [^1^A]_0_/[^1^S]_0_ ratios will in fact be competing with the ubiquitous inter-UC pathway. If the efficiency of the intra-UC is lower than that of inter-UC, this would then be detrimental to UC performance, as indicated by our results.

While these results are interesting on their own, one must consider in what settings intra-UC is anticipated to be of importance. As solid-state solutions are a desirable path moving forward, it is in the context of restricted molecular diffusion these results must be interpreted.^19,56−61^ With respect to this, it is improbable that the TETA mechanism will be active in future solid-state systems where interactions between multichromophoric annihilators are hampered. These implications are strengthened by the results from the study by Dzebo et al., where large DPA annihilator frameworks showed better UC performance than DPA when put inside a rigid polymer matrix.^23^ First, the performance of these compounds improved with molecular (dendrimeric/oligomeric) size and even outperformed a reference sample consisting of the DPA monomer, despite the fact that the molecular concentration of DPA was almost 10 times higher in the reference sample than in the oligomeric frameworks. Second, it was found that the best performance was achieved when the sensitizer concentration was on the same order as that of the annihilator. Together, this forms unequivocal proof that it is the DS mechanism that is active in such systems and that intra-UC in fact may enhance UC performance in solid-state systems. Major challenges in facilitating the energy transfer events that are needed remain, particularly how to precisely control the spatial association and interactions between the sensitizer and annihilator. Efforts to understand and control the complicated assembly of high-performing solid-state UC systems are currently undergoing in our lab.

## Conclusions

In this study, four novel dimers based on DPA have been synthesized and evaluated as annihilators for TTA-UC. The upconversion efficiencies of these molecules are high throughout, with the meta-coupled 1,3-DPA_2_ in particular performing on par with DPA. This is primarily ascribed to the long triplet lifetime of this dimer, which is at least five times longer than that of the other dimers and similar to the triplet lifetime of DPA. The importance of the triplet lifetime to achieve efficient TTA-UC is thus reinforced, and a facile method to accurately measure this important parameter has been utilized. Furthermore, the mechanism of intramolecular upconversion has been investigated by comparison between simulations and experiments. Our results from time-resolved UC emission measurements firmly ascertain that the DS mechanism is active in systems with dimeric annihilators and that this additional pathway could be of great importance under specific conditions, e.g., at extremely low annihilator to sensitizer concentration ratios or in diffusionally restricted media. While the full picture of intra-UC is still lacking, especially in terms of how efficiently intra-UC may proceed, our results may act as a guide for future solid-state designs where exciton migration is envisioned as a crucial component.

## Supporting Information Available

Detailed description of the synthesis of novel compounds, proton and carbon NMR spectra, descriptions of experimental setups, additional spectroscopic and modeling data, full model descriptions, and full details on the temperature-dependent photophysics of 1,2-DPA_2_ (PDF)
