# Supplementary material for: Diphenylanthracene Dimers for Triplet–Triplet Annihilation Photon Upconversion: Mechanistic Insights for Intramolecular Pathways and the Importance of Molecular Geometry
Source: J Am Chem Soc. 2021 Apr 9;143(15):5745–54. doi: 10.1021/jacs.1c00331 (PMC8154513; doi:10.1021/jacs.1c00331)
Supplement: Supplementary file 1 — ja1c00331_si_001.pdf [file ja1c00331_si_001.pdf]

Supporting Information (SI) for:

Diphenylanthracene dimers for triplet-triplet annihilation photon upconversion: mechanistic insights for intramolecular pathways and the importance of molecular geometry

Axel Olesund[†], Victor Gray[†,§], Jerker Mårtensson[†], and Bo Albinsson*[,†]

[†]Department of Chemistry and Chemical Engineering, Chalmers University of Technology, 412 96 Gothenburg, Sweden
[§]Department of Chemistry, Ångström Laboratory, Uppsala University, Box 523, 751 20 Uppsala, Sweden

Table of Contents

1. Synthesis of annihilator compounds 2
1.1 9,9'-PA_2 3
1.2 1,2-DPA_2 3
1.3 1,3-DPA_2 4
1.4 1,4-DPA_2 4

2. Experimental methods 9
2.1 Instrumentation and optical measurements 9
2.2 Quantum mechanical calculations 9

3. Excimer formation in 1,2-DPA_2 10
3.1 Photophysical characterization 10
3.2 Modelling and fitting 12

4. Photophysical characterization 16
4.1 Upconversion measurements 16
4.2 Determination of triplet excited state lifetimes 17
4.3 Determination of the triplet-triplet annihilation rate constant 23
4.4 Calculations on electronic coupling of triplet excited states 26

5. Intra-molecular upconversion: Rate equations, simulations, and measurements 27
5.1 Model descriptions 27
5.2 Time-resolved simulations 28
5.3 Steady-state simulations 29
5.4 Settings and parameters 30
5.5 Simulation and experimental results 31

6. References 36

1. Synthesis of annihilator compounds

Starting materials were purchased from Sigma-Aldrich or Fisher Scientific and used as received if not stated otherwise. Dry THF was obtained by distilling over sodium metal. NMR was run on a 400 MHz Varian NMR. IR was run on a Perkin Elmer ATR-FTIR. Column chromatography was either carried out using a Biotage Flash Column Chromatography system with Biotage prepacked SNAP columns or manually over silica gel. High-resolution mass spectroscopy (HRMS) was performed by CMSI at Chalmers University of Technology. HRMS was carried out on an Agilent 1290 Infinity LC system equipped with an autosampler tandem to an Agilent 6520 Accurate Mass Q-TOF LC/MS. HRMS was analysed without a column, with a 0.4 mL/min flow rate using an isocratic method (50% MPA/50% MPB), with a Mobile Phase A (MPA): 50% water/50% MeOH and Mobile Phase B (MPB): MeOH.

The preparation of 9-bromo-10-phenylanthracene and 4,4,5,5-tetramethyl-2-(10-phenylanthracen-9-yl)-1,3,2-dioxaborolane has been reported previously.[1] 9,9'-PA$_2$ was synthesized according to literature procedures starting with the reductive

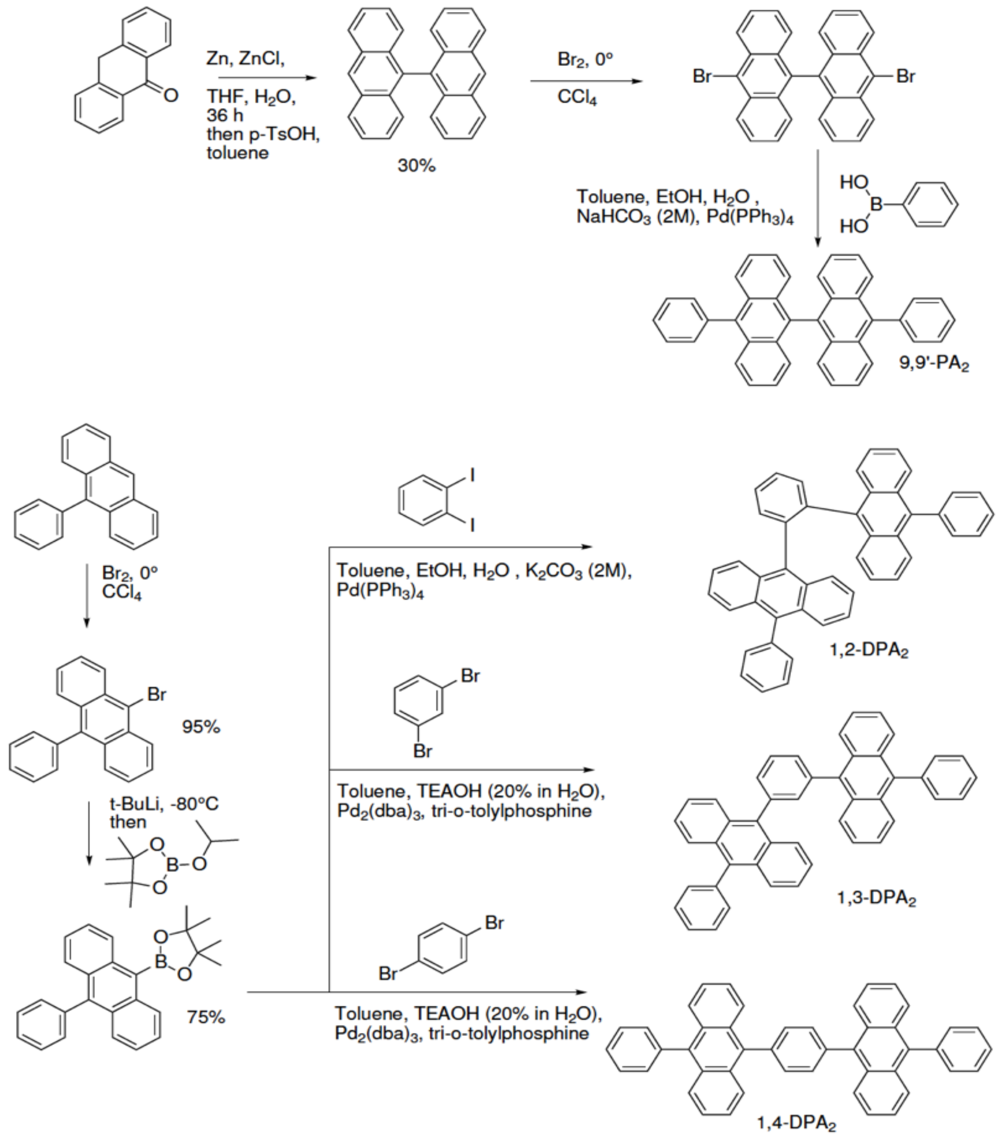

Figure S1: Synthetic route of diphenylanthracene dimers.

dimerization of anthrone, followed by dehydration using p-toluenesulfonic acid.[2] 9,9-bianthracene was brominated with elemental bromine[2] and 9,9'-PA_2 was afforded through a Suzuki cross-coupling reaction with phenylboronic acid, as described below. The synthesis of 1,2-DPA_2 was based on the procedures reported by Nishiuchi et al. for 1,2-di(9-anthryl)benzene.[3] NMR spectra for each compound is found after the synthesis description.

1.1 9,9'-PA_2

908 mg of 10,10'-dibromo-9,9'-bianthracene (1.8 mmol) was added to a microwave vial together with 1.00 g of phenylboronic acid (8.2 mmol) and 360 mg $Pd(PPh_3)_4$ (0.31 mmol, 17%). The vial was sealed and the atmosphere was exchanged to argon. Then 20 ml toluene and 8 ml ethanol were added together with 4 ml aqueous $NaHCO_3$ (2 M). All liquids were purged with argon for 30 minutes before addition. The mixture was refluxed at 100°C for 5h. The crude was extracted with toluene, washed with water and brine, and the organic fraction was evaporated to dryness. The crude was loaded onto a SNAP biotage column and eluted with petroleum ether with increasing DCM content, 0-20%. The obtained product was further recrystallized from toluene to yield a pale white powder, 215 mg (Yield: 24%). NMR spectra are in good agreement to that reported previously.[4] ^1H NMR (400 MHz,$CDCl_3$) δ = 7.83 (dt, J_1 = 8.8 Hz, J_2 = 0.9 Hz, 4H) 7.70 - 7.59 (m, 10H), 7.35 (dd, J_1 = 6.3 Hz, J_2 = 1.3 Hz, 2H), 7.32 (dd, J_1 = 6.3 Hz, J_2 = 1.3 Hz, 2H), 7.26 (dt, J_1 = 8.9 Hz, J_2 = 1.0 Hz, 4H) 7.18 (dd, J_1 = 6.3 Hz, J_2 = 1.2 Hz, 2H), 7.15 (dd, J_1 = 6.3 Hz, J_2 = 1.2 Hz, 2H).
^{13}C NMR (100 MHz, $CDCl_3$) δ = 139.05, 137.84, 133.38, 131.44, 131.34, 130.06, 128.45, 127.55, 127.22, 127.07, 125.50, 125.19.

FT-IR (ATR) ν (cm^{-1}) 3076 (bw), 3012 (bw), 1599 (w), 1518 (w), 1498 (w), 1441 (m), 1359 (m), 1154 (w), 1068 (w), 1022 (m), 1000 (w), 934 (m), 838 (m), 777 (s), 768 (s), 705 (s), 676 (s), 672 (s), 646 (w), 612 (m).
HRMS ($(m+H)^+/z$): Calculated for $C_{40}H_{26}$ = 507.2107, found 507.2112.
Elemental Analysis: Calculated for $C_{40}H_{26}$, C: 94.83 %, H: 5.17 %, found C: 94.53 %, H: 5.33 %.

1.2 1,2-DPA_2

1,2-diiodobenzene (0.11 ml, 0.9 mmol) was added together with 4,4,5,5-tetramethyl-2-(10-phenylanthracen-9-yl)-1,3,2-dioxaborolane (1.00 g, 2.6 mmol), $Pd(PPh_3)_4$ (202 mg, 0.18 mmol, 10%) and K_2CO_3 (360 mg, 2.6 mmol) to a microwave vial. The atmosphere was exchanged for argon and then toluene (8.8 ml), ethanol (1.5 ml) and water (1.5 ml), all purged with argon for 30 minutes, were added. The mixture was refluxed at 110°C for 65 h. The reaction mixture was extracted with toluene, washed with water and brine, and the organic fractions were evaporated to dryness. The crude was loaded onto a SNAP biotage column and eluted with petroleum ether with increasing DCM content, 0-20%. The third, yellow fraction was collected, and the product was further recrystallized from DCM and methanol to yield yellow crystals, 66 mg (Yield: 26%). ^1H NMR (400 MHz, $CDCl_3$) δ = 7.97 - 7.93 (m, 2H), 7.88 - 7.84 (m, 2H), 7.81 (d, J = 8.7 Hz, 4H), 7.48-7.39 (m, 6H), 7.26 - 7.24 (m, 2H), 7.22 (d, J = 8.7 Hz, 4H), 7.01 (dd, J_1 = 6.4 Hz, J_2 = 1.2 Hz, 2H), 6.97 (dd, J_1 = 6.4 Hz, J_2 = 1.2 Hz, 2H), 6.91 (dd, J_1 = 6.4 Hz, J_2 = 1.2 Hz, 2H), 6.87 (dd, J_1 = 6.4 Hz, J_2 = 1.2 Hz, 2H), 6.82 - 6.78 (m, 2H). ^{13}C NMR (100 MHz, $CDCl_3$) δ = 140.23, 139.04, 136.12, 135.40, 133.16, 131.03, 130.93, 129.37, 128.97, 128.09, 127.99, 127.60, 127.31, 127.08, 126.09, 124.20, 123.74.

FT-IR (ATR) ν (cm^{-1}) 3058 (bw), 3025 (bw), 1604 (w), 1493 (w), 1445 (bw), 1384 (s), 1159 (w), 1134 (w), 1093 (w), 1078 (w), 1027 (w), 1002 (w), 990 (w), 939 (m), 767 (s), 758 (s), 715 (m), 706 (s), 651 (s), 632 (m), 613 (m).

HRMS ((m+H)$^+$/z): Calculated for $C_{46}H_{30}$ = 583.2420, found 583.2432
Elemental Analysis: Calculated for $C_{46}H_{30}$, C: 94.81 %, H: 5.19 %, found C: 94.64 %, H: 5.26 %.

1.3 1,3-DPA$_2$

1,3-dibromobenzene (0.14 ml, 1.1 mmol) was added together with 4,4,5,5-tetramethyl-2-(10-phenylanthracen-9-yl)-1,3,2-dioxaborolane (1.28 g, 3.4 mmol), $Pd_2(dba)_3$ (31 mg, 0.03 mmol, 1.5%) and tri-*o*-tolylphosphine (41 mg, 0.13 mmol) to a microwave vial.
The atmosphere was exchanged for argon and then toluene (15 ml) and tetraethylammonium hydroxide (12 ml, 20% in water), both purged with argon for 30 minutes, were added.
The mixture was refluxed at 110°C for 40 h. The reaction mixture was extracted with toluene, washed with water and brine, and the organic fractions were evaporated to dryness. The crude was loaded onto a SNAP biotage column and eluted with petroleum ether with increasing DCM content, 0-40%. The third, yellow fraction was collected and the product was further recrystallized from DCM and methanol to yield yellow crystals, 519 mg (Yield: 79%). ^{1}H NMR (400 MHz, $CDCl_3$) δ = 8.01 (dt, J_1 = 8.7 Hz, J_2 = 1.2 Hz, 4H), 7.87 (t, J = 7.3 Hz, 1H), 7.72, - 7.69 (m, 6H), 7.66 - 7.50 (m, 9H), 7.46 - 7.42 (m, 6H), 7.35 (dd, J_1 = 6.5 Hz, J_2 = 1.3 Hz, 2H), 7.33 (dd, J_1 = 6.5 Hz, J_2 = 1.3 Hz, 2H). ^{13}C NMR (100 MHz, $CDCl_3$) δ = 139.18, 139.01, 137.27, 136.69, 134.50, 131.32, 131.26, 130.60, 129.90, 128.50, 128.39, 128.37, 127.45, 127.05, 126.87, 125.18, 124.99.

FT-IR (ATR) ν (cm^{-1}) 3063 (bw), 3030 (bw), 1599 (w), 1500 (w), 1440 (m), 1384 (m), 1372 (w), 1174 (w), 1147 (w), 1065 (w), 1025 (m), 1000 (w), 940 (w), 921 (w), 810 (m), 770 (s), 760 (s), 711 (w), 701 (s), 666 (m), 659 (m), 638 (m), 618 (m), 605 (m).
HRMS ((m+H)$^+$m/z): Calculated for $C_{46}H_{30}$ = 583.2420, found 583.2416.
Elemental Analysis: Calculated for $C_{46}H_{30}$, C: 94.81 %, H: 5.19 %, found C: 94.05 %, H: 5.39 %.

1.4 1,4-DPA$_2$

1,4-dibromobenzene (125 mg, 0.53 mmol) was added together with 4,4,5,5-tetramethyl-2-(10-phenylanthracen-9-yl)-1,3,2-dioxaborolane (600 mg, 1.58 mmol), $Pd_2(dba)_3$ (20 mg, 0.02 mmol, 1.5%) and tri-*o*-tolylphosphine (35 mg, 0.08 mmol) to a microwave vial. The atmosphere was exchanged for argon and then toluene (8 ml) and tetraethylammonium hydroxide (8 ml, 20% in water), both purged with argon for 30 minutes, were added. The mixture was refluxed at 110°C for 16 h. The reaction was quenched with water, and then filtered. The filtrate was washed with ethanol and water. The product was further recrystallized from DCM and ethanol to yield a pale white powder, 121 mg (Yield: 40%). ^{1}H NMR (400 MHz, $CDCl_3$) δ = 7.99 (dt, J_1 = 0.8 Hz, J_2 = 8.7 Hz, 4H), 7.77 (dt, J_1 = 1.1 Hz, J_2 = 8.4 Hz, 4H), 7.74 (s, 4H), 7.67 - 7.63 (m, 4H), 7.61 - 7.53 (m, 6H), 7.50 (dd, J_1 = 1.3 Hz, J_2 = 6.4 Hz, J_3 = 8.8 Hz, 4H), 7.41 (dd, J_1 = 1.3 Hz, J_2 = 6.4 Hz, J_3 = 8.8 Hz, 4H). ^{13}C NMR (100 MHz, $CDCl_3$) δ = 139.07, 138.23, 136.87, 131.41, 131.34, 130.00, 128.43, 127.50, 127.10, 126.99, 125.28, 125.22, 125.10.

FT-IR (ATR) ν (cm^{-1}) 3068 (bw), 3020 (bw), 1599 (w), 1515 (w), 1495 (w), 1440 (m), 1386 (m), 1174 (w), 1070 (w), 1030 (m), 943 (m), 830 (w), 767 (s), 755 (s), 727 (m), 702 (s), 694 (m), 656 (s), 638 (w), 621 (w), 612 (m), 539 (w).
HRMS ((m+H)$^+$/z): Calculated for $C_{46}H_{30}$ = 583.2420, found 583.2418
Elemental Analysis: Calculated for $C_{46}H_{30}$, C: 94.81 %, H: 5.19 %, found C: 94.63 %, H: 5.30.

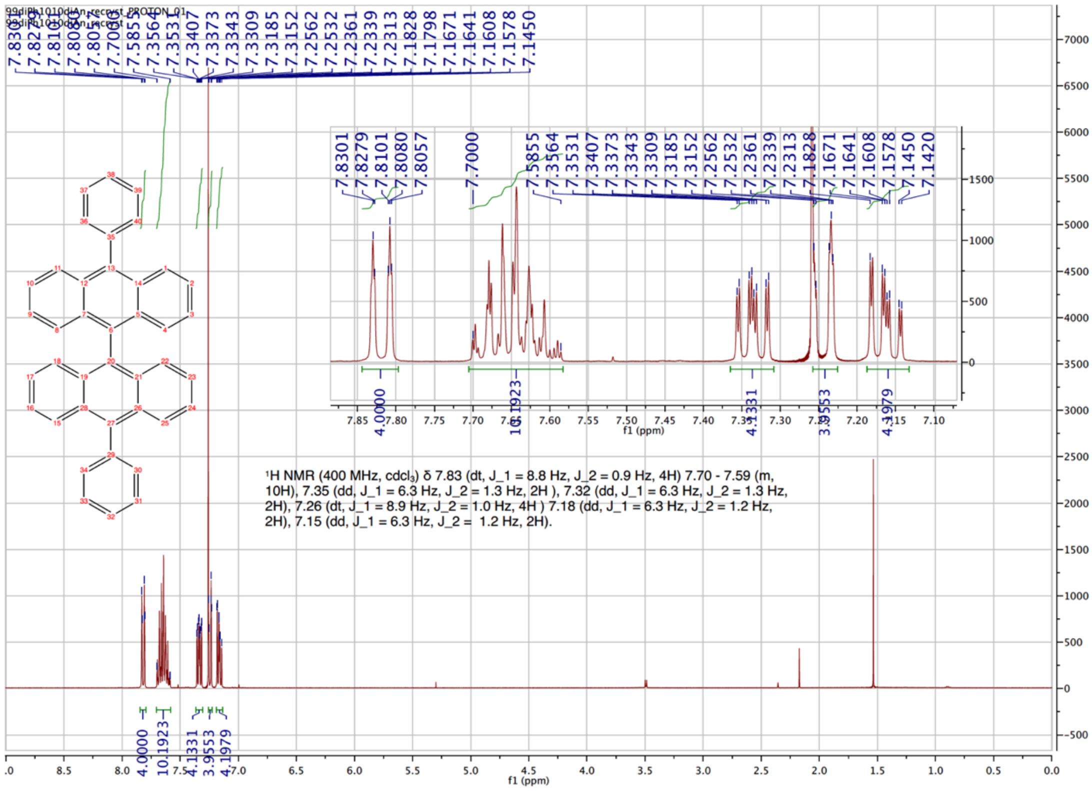

Figure S2. ^{1}H-NMR spectrum of 9,9'-PA$_2$.

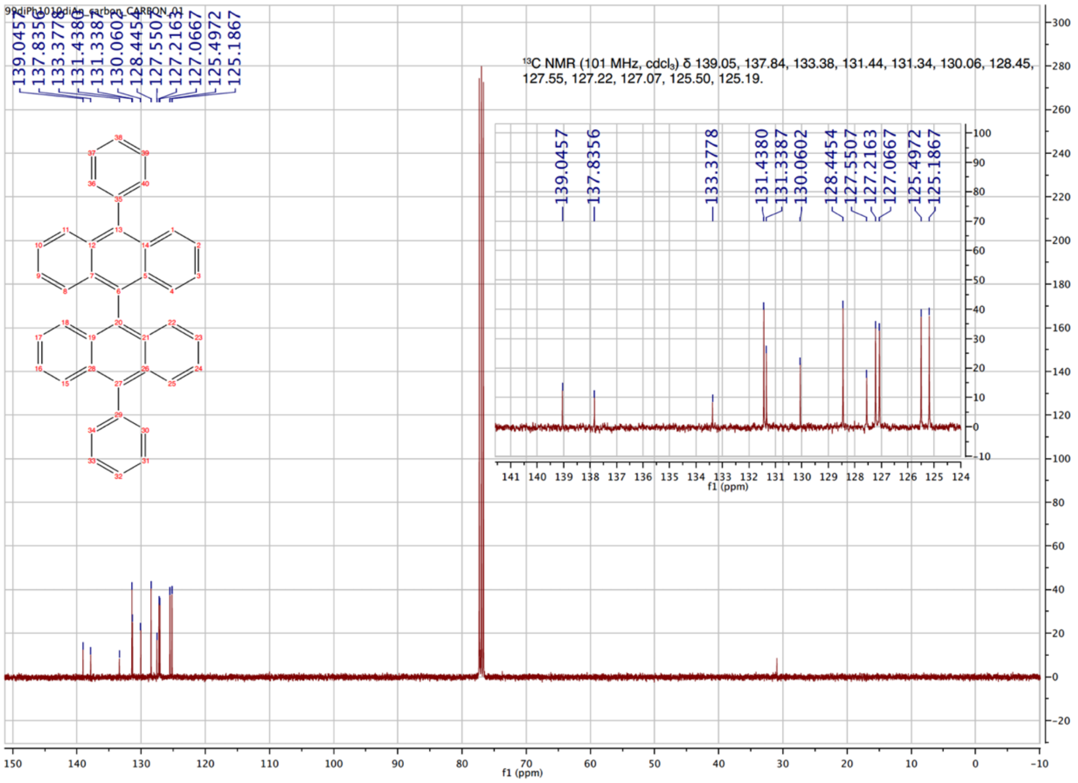

Figure S3. ^{13}C-NMR spectrum of 9,9'-PA$_2$.

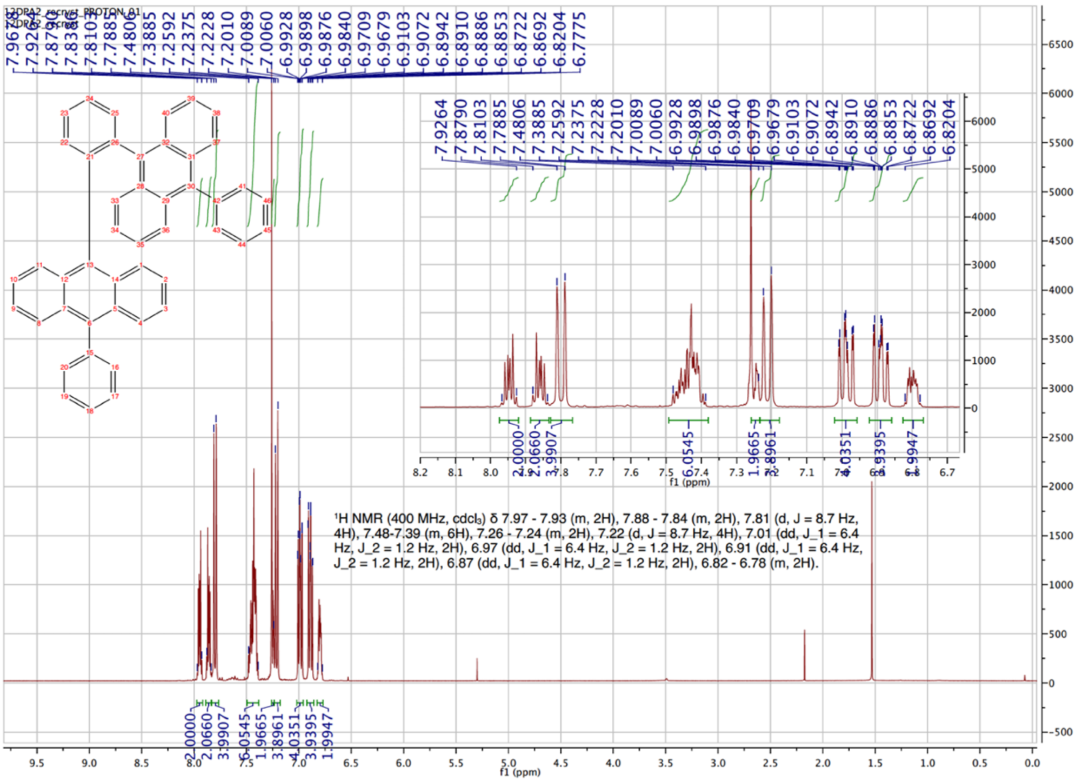

Figure S4. ^{1}H-NMR spectrum of 1,2-DPA$_2$.

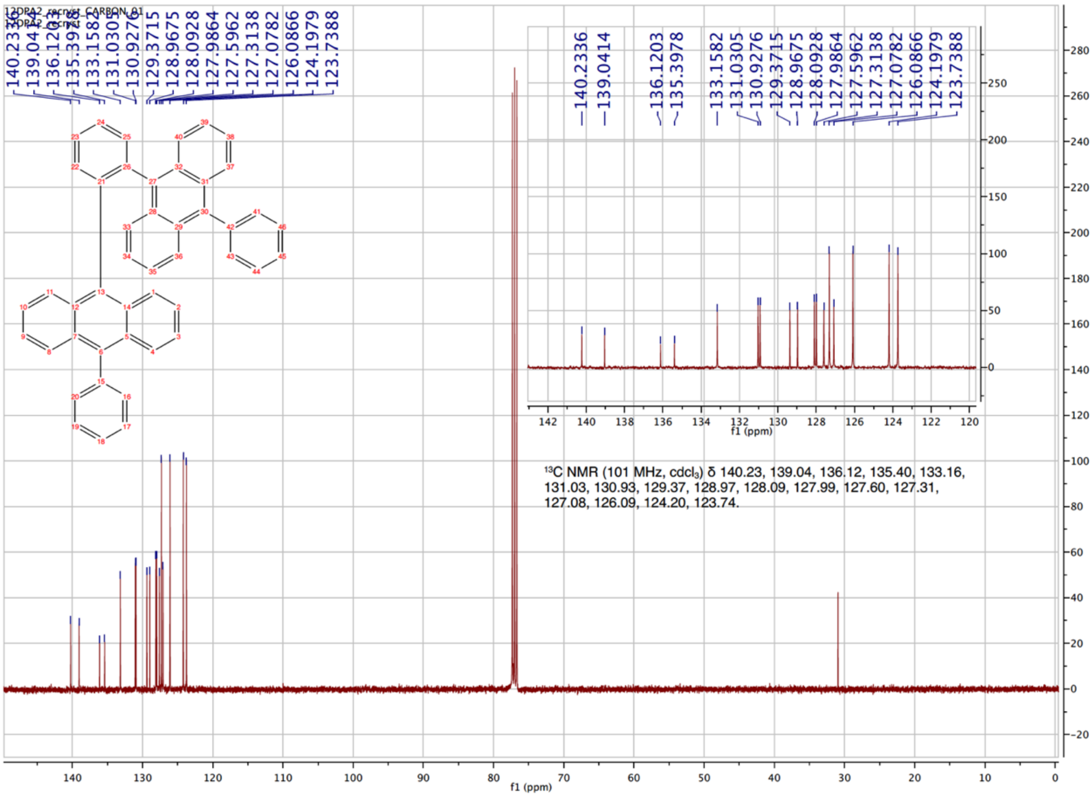

Figure S5. ^{13}C-NMR spectrum of 1,2-DPA$_2$.

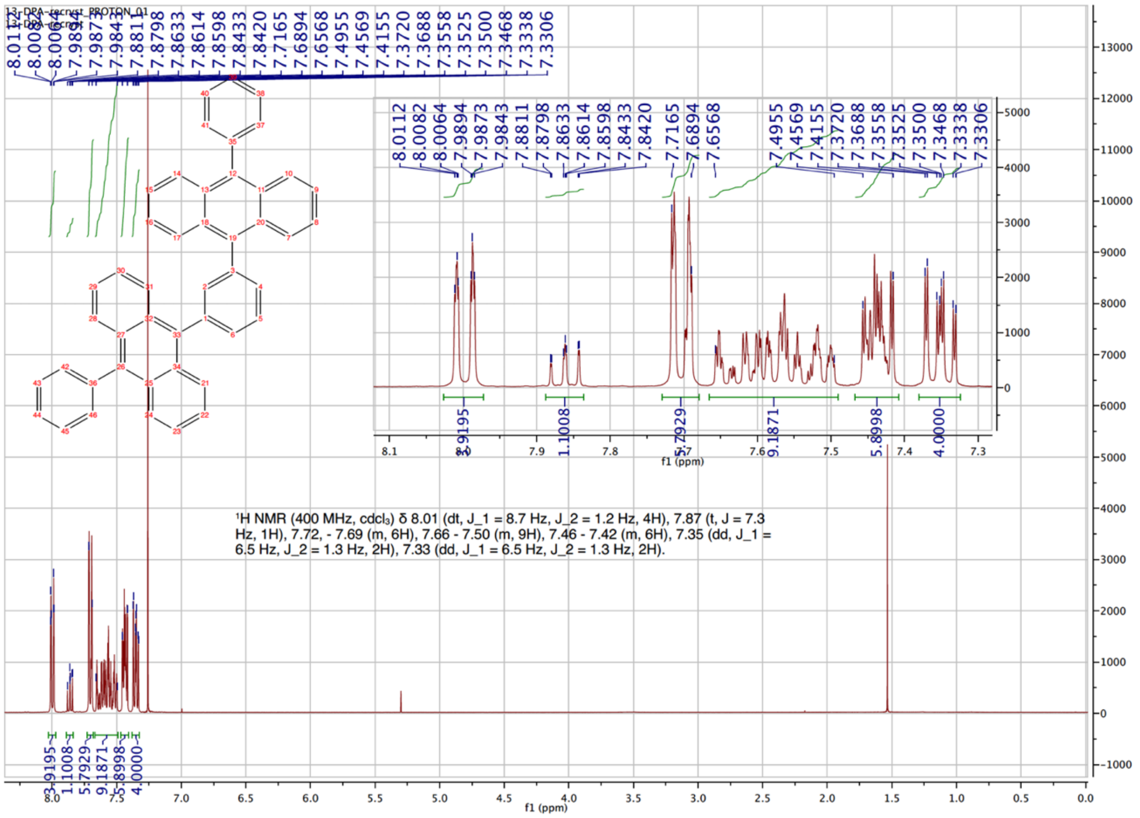

Figure S6. ^{1}H-NMR spectrum of 1,3-DPA$_2$.

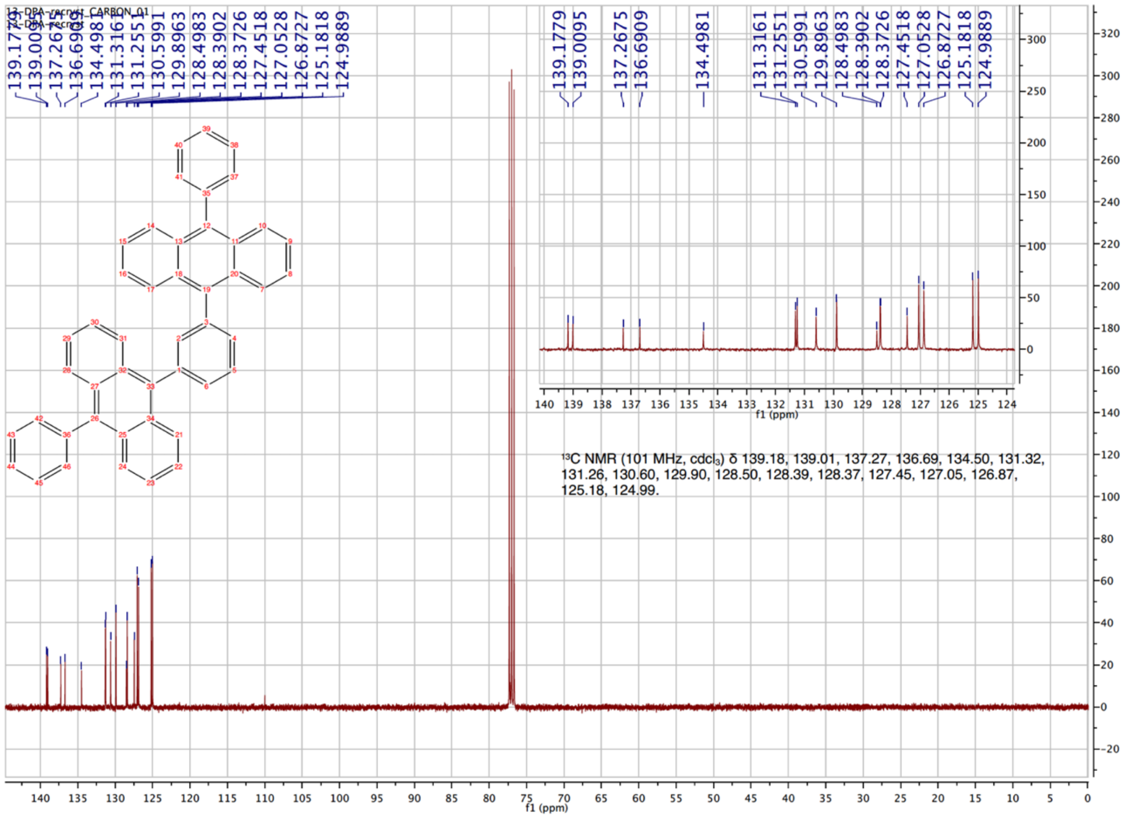

Figure S7. ^{13}C-NMR spectrum of 1,3-DPA$_2$.

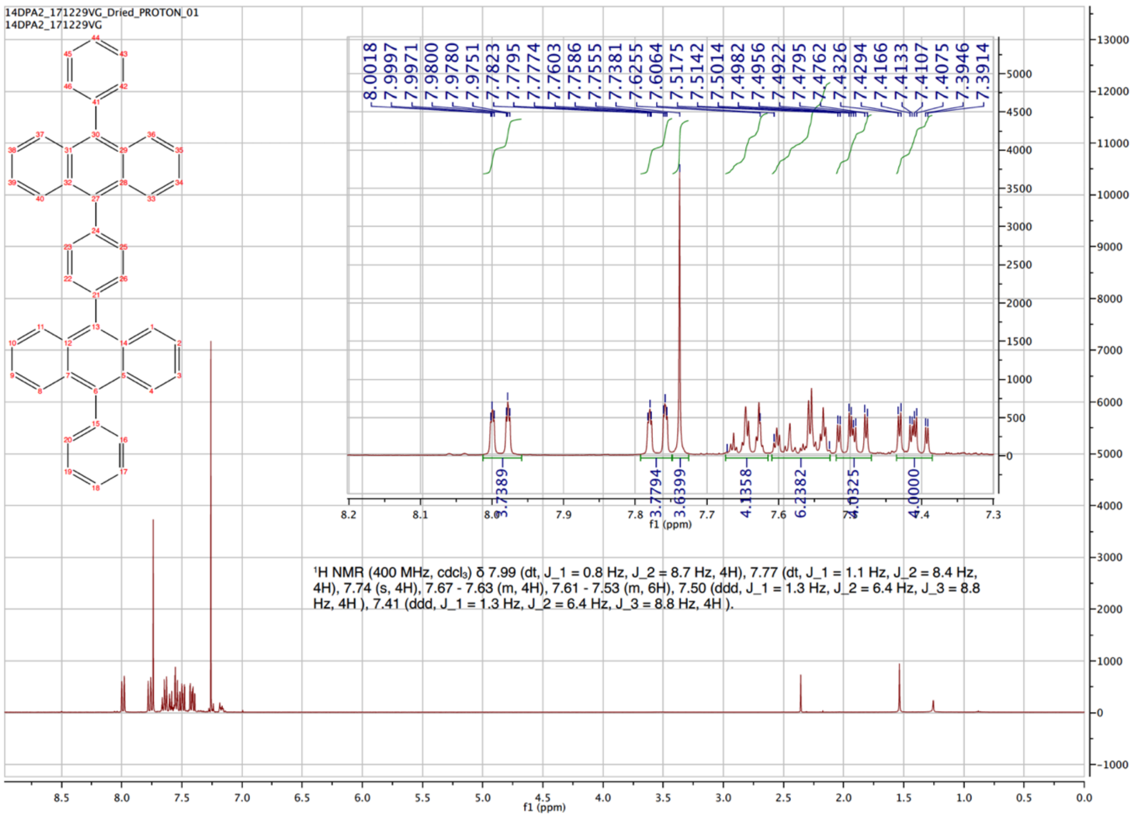

Figure S8. ^{1}H-NMR spectrum of 1,4-DPA$_2$.

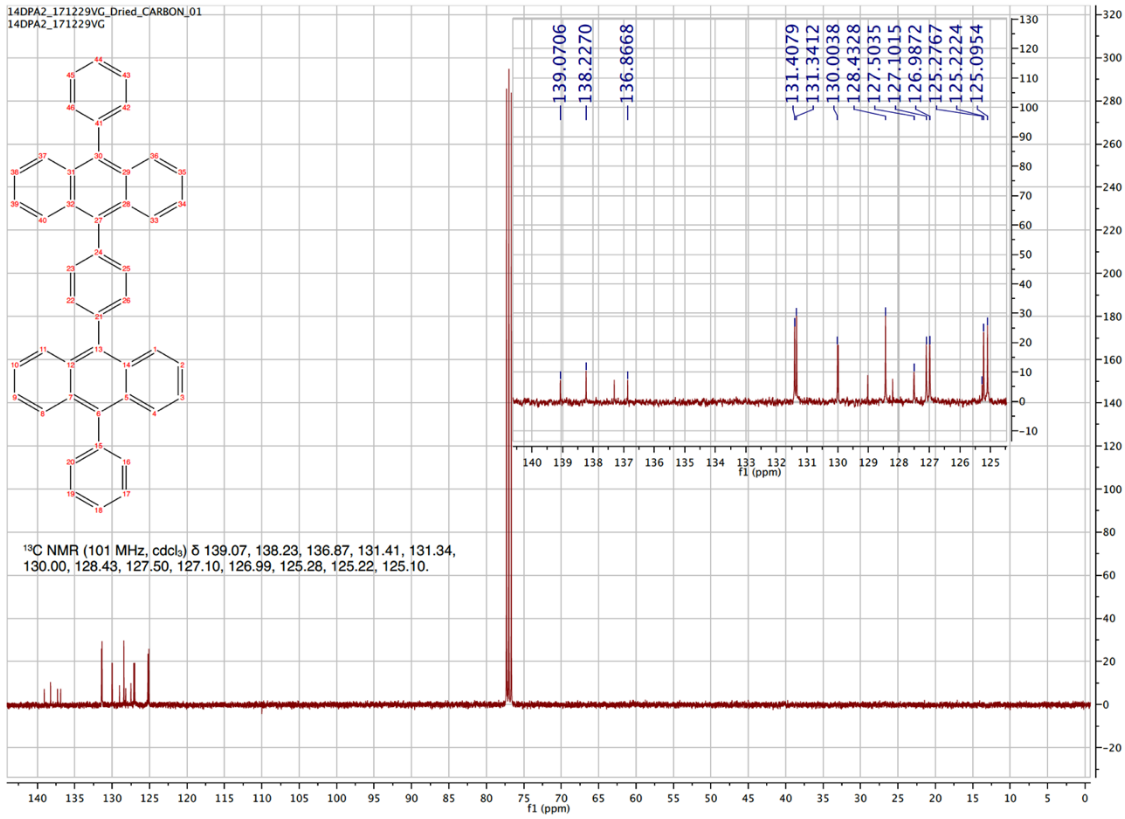

Figure S9. ^{13}C-NMR spectrum of 1,4-DPA$_2$.

2. Experimental methods

2.1 Instrumentation and optical measurements

Steady-state absorption spectra were recorded on a Cary 50 UV-vis-NIR spectrophotometer and steady-state fluorescence measurements were carried out on a Spex Fluorolog 3 spectrofluorometer (Horiba Jobin Yvon). Fluorescence lifetimes were determined on a time correlated single photon counting (TCSPC) setup using PicoQuant laser diodes (377 nm) and a PMT detector (10 000 counts, 4096 channels). Steady-state upconversion fluorescence measurements were performed on a home-built system using a continuous-wave 532 nm OBIS laser (Coherent) as the excitation source. The measured maximum power output was 98 mW and the laser beam diameter 0.7 mm. A linear variable neutral density (ND) filter was used to vary the laser intensity, and data were recorded using home-built LabView software. Nanosecond transient absorption measurements were performed on a home-built system using a Nd:YAG (Spectra Physics, Quanta-Ray) laser equipped with an OPO (Spectra Physics, primoScan) generating a 10 ns pump beam. A quartz-halogen lamp was used as the probe light and a monochromator together with a 5-stage PMT coupled to an oscilloscope was used for recording the transient. All photophysical measurements were carried out in toluene using 10 mm quartz cuvettes except for samples for upconversion, which were carried out using 2 mm (steady-state UC) or 4 mm (time-resolved UC) cuvettes to minimize the influence of secondary inner-filter effects. All samples were prepared in a nitrogen glovebox (Innovative Technologies) with <0.1 ppm oxygen levels and sealed with air-tight cap screws and parafilm. Temperature-dependent measurements were performed using a liquid nitrogen cryostat (Oxford Instruments) connected to a temperature controller.

Fluorescence quantum yields were determined using relative actinometry, utilizing deoxygenated DPA in cyclohexane ($\phi_F = 1.00$)[5] as the reference compound.

2.2 Quantum mechanical calculations

Density functional calculations (DFT) were performed using the Gaussian 16 software package[6] using the hybrid functional B3LYP and the basis set 6-31G(2d,p). Full optimization of the ground-state structure was followed by excited state calculations using the same basis set. Excited state energies (20 lowest) were calculated using the time-dependent formalism (TDDFT) and vertical excitation energies for the lowest electronic transitions are given in Table 2 of the main text and compared to experiments. There is an excellent agreement between experiments and calculations with only a small blue shift (average 0.14 eV) of the calculated singlet transitions ascribed to solvent shifts and inherent inaccuracy of the theoretical methods. For 1,2-DPA_2 and 9,9'-PA_2 the excited state energy calculations were very sensitive to minor changes in the structures, most likely reflecting the comparably high electronic coupling between the DPA moieties in these two dimers. A few calculations using a triple zeta basis set (6-311G(2d,p)) were done on DPA resulting in minute differences in the excited state energies.

3. Excimer formation in 1,2-DPA$_2$

3.1 Photophysical characterization

Figure S10 shows the absorption spectra of 1,2-DPA$_2$ in 2-methyltetrahydrofuran (MeTHF) at different temperatures. MeTHF was used as the solvent as it has a glassing transition at circa 135 K, allowing for low-temperature optical measurements. The absorptive transitions remain at the same positions for the entire temperature range, demonstrating that no inter- or intramolecular interactions take place in the ground state.

Figure S11 shows the temperature-dependent emission from 1,2-DPA$_2$ in MeTHF. There is a transition from long wavelength (475 nm to 650 nm) fluorescence originating from the excimer form (E) to a short wavelength, highly structured fluorescence similar to the fluorescence of the other dimers. At high T only weak fluorescence is detected from this "monomeric" excited state (M) where the two chromophore moieties do not interact with each other. The transition from E-type fluorescence to M-type fluorescence starts at approx. 175 K, and as 113 K is reached the initial formation of the E state more or less disappears.

To determine the quantum yield of the E state fluorescence, an asymmetric Gaussian fitting function was used (see Figure S12). The M state fluorescence was then calculated as the difference between total fluorescence and the fitted E state fluorescence.

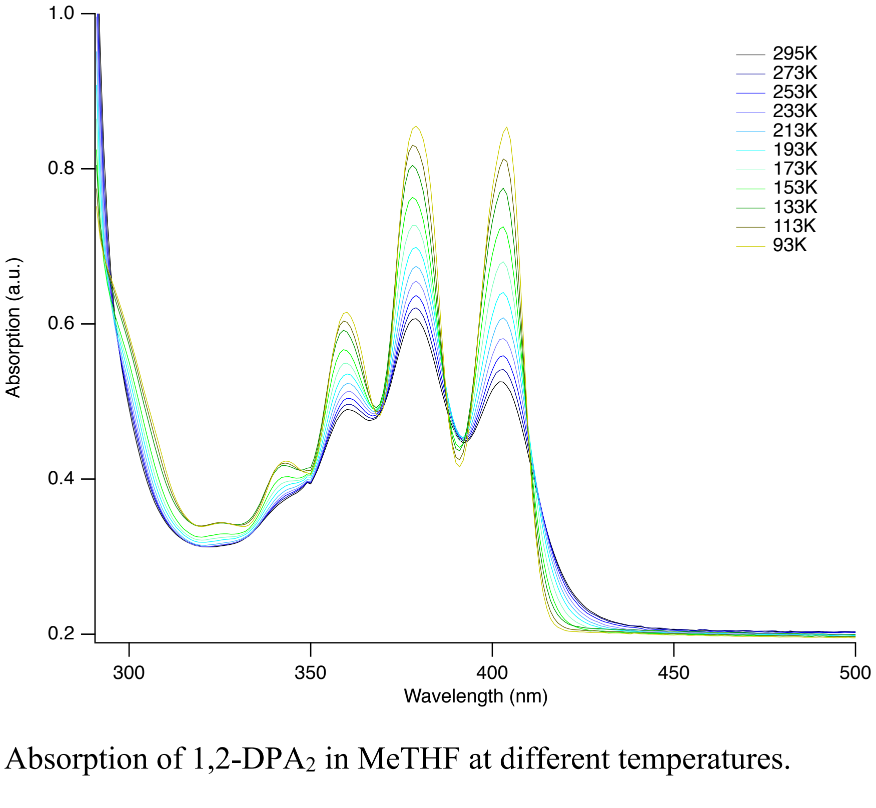

Figure S10. Absorption of 1,2-DPA$_2$ in MeTHF at different temperatures.

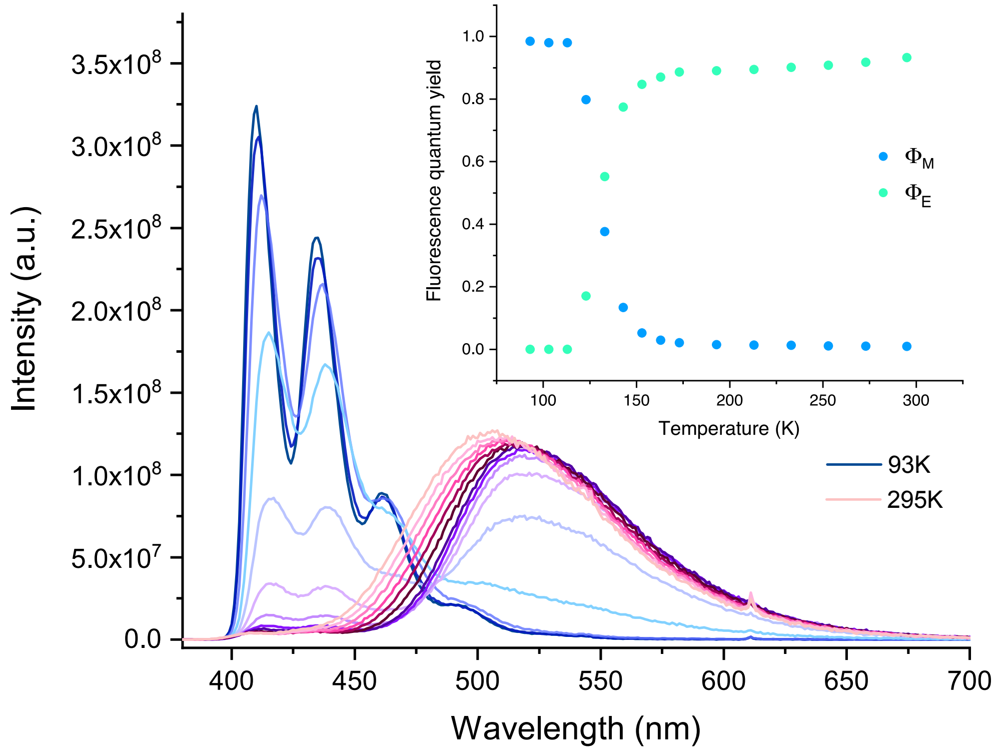

Figure S11. Temperature dependent fluorescence of 1,2-DPA$_2$ in deoxygenated MeTHF. *Inset:* fluorescence quantum yield for the monomeric (M) and excimeric (E) states at different temperatures.

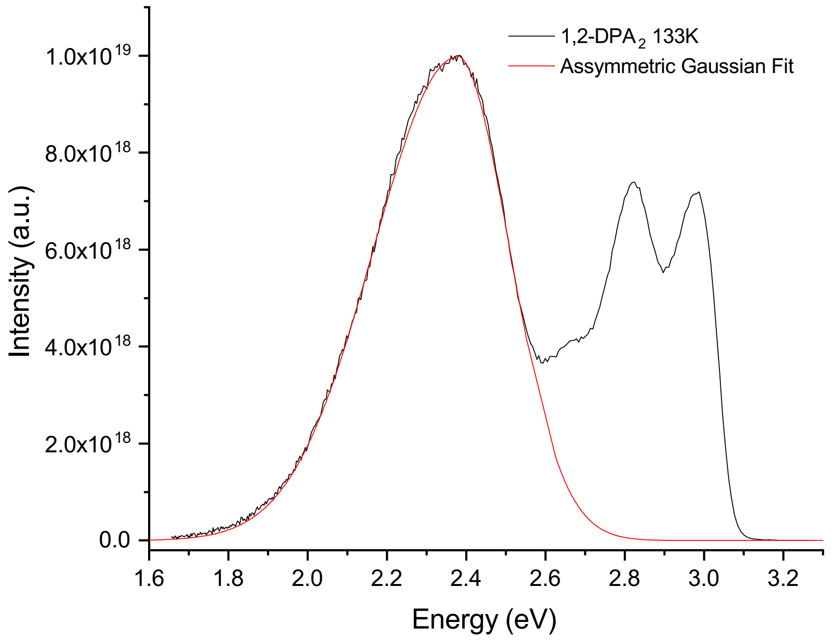

Figure S12. Example of how the excimer fluorescence is fitted to a asymmetric Gaussian function for E state fluorescence quantum yield determination.

3.2 Modelling and fitting

The expressions below are the definitions of quantum yield (ϕ) and lifetime (τ) of a process from a state in a molecule:

$$\phi = \frac{k_f}{k_f + \sum_i k_i}$$

$$\tau = \frac{1}{k_f + \sum_i k_i}$$

$$\rightarrow k_f = \frac{\phi}{\tau}$$

where k_f is the rate constant for fluorescence. In this case (two-state model, see Figure S13) we can make the assumption that:

$$\phi_M(T \rightarrow 0) = 1 \rightarrow k_{nr,M} \approx 0$$

where ϕ_M is the fluorescence quantum yield of the "monomeric" singlet excited state. The quantum yield at low temperature has been shown to be close to unity, see Table S1.

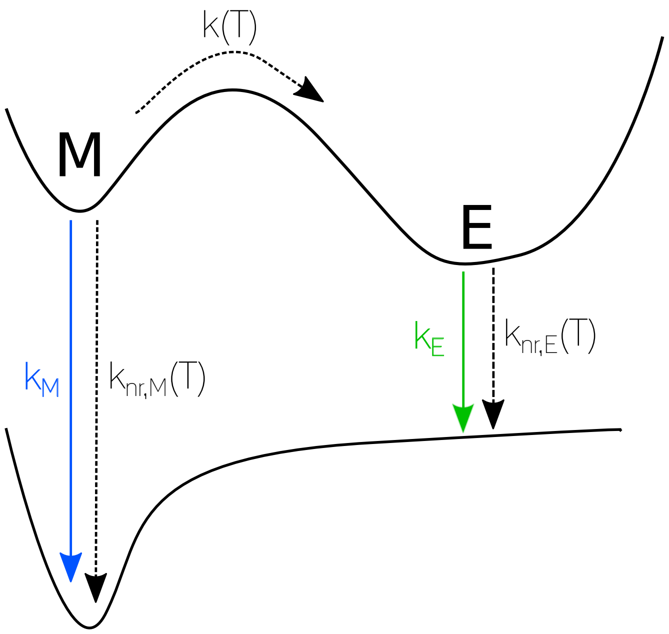

Figure S13. Model description of the intramolecular excimer (E) formation in 1,2-DPA$_2$.

Two of the rate constants (k(T) and $k_{nr,E}$(T)) are assumed to follow Arrhenius behavior. By performing quantum yield measurements (giving ϕ_M and ϕ_E) and lifetime measurements (giving τ_M and τ_E) four unknowns remain (k_M, k, k_E, and $k_{nr,E}$), and the equation system below can be solved.

$$\phi_M(T) = \frac{k_M(T)}{k_M(T)+k(T)} \tag{E1}$$

$$\phi_E(T) = C_E \frac{k_E(T)}{k_E(T)+k_{nr,E}(T)} \;\; ; \; C_E = \frac{k(T)}{k(T)+k_M(T)} \tag{E2}$$

$$\frac{\phi_M(T)}{\tau_M(T)} = k_M(T) \tag{E3}$$

$$\frac{1}{k_E(T)+k_{nr,E}(T)} = \tau_E(T) \tag{E4}$$

Combining these equations, the following expressions are obtained:

$$\text{(E1)} + \text{(E3)} \rightarrow k(T) = \frac{1-\phi_M(T)}{\tau_M(T)} = k_0 \exp\left(-\frac{E_a}{RT}\right) \tag{E5}$$

$$(E3) \rightarrow \frac{\phi_M(T)}{\tau_M(T)} = k_M(T) \quad (E6)$$

$$(E2) + (E4) \rightarrow \frac{\phi_E(T)}{\tau_E(T)}(1 + \frac{\phi_M(T)}{1-\phi_M(T)}) = k_E(T) \quad (E7)$$

$$(E4) \rightarrow k_{nr,E}(T) = \frac{1}{\tau_E(T)} - k_E(T) = k_{nr,0} \exp(-\frac{E_{nr,a}}{RT}) \quad (E8)$$

The radiative rate constants k_E and k_M are, as usual, expected to be independent of temperature, and the current model indicates a good degree of temperature independence for k_E, but not as well for k_M. This is probably due to uncertainty in the M state lifetime at higher T. In the model, a set value of $k_M = 1.33*10^8$ s^{-1} (corresponding to a M state lifetime of ca 7.5 ns) is used. For the M state values in the temperature range 93K to 233K are used. This is because the M state lifetimes are too short to be measured with our TCSPC setup at higher T. For the E state the temperature range 123K to 295K is used. Below 123K the excimer state isn't sufficiently populated in order to yield a detectable signal for lifetime determination. Even though k_E has a low temperature dependence, this dependence is stronger than that for the excimer lifetime, yielding higher non-radiative rate constants at low temperature if used, something that would be physically contradictory (see Equation E7). During fitting procedures, k_E has been set to a constant value of $1.97*10^7$ s^{-1}, which is the mean of the temperature-dependent values achieved during measurements.

Fitting the data to an Arrhenius equation gives the following parameters to current data:

k_0 = $2.1*10^{14}$ s^{-1} (pre-exponential factor for barrier between monomer and excimer, see expression for k(T) above).

E_a = $1.3*10^4$ J mol^{-1} (activation energy for barrier between monomer and excimer)

$k_{nr,0}$ = $4.7*10^6$ s^{-1} (pre-exponential factor for non-radiative decay from excimeric state, see expression for $k_{nr,E}$(T) above).

$E_{a,nr}$ = $1.2*10^3$ J mol^{-1} (activation energy for non-radiative decay from excimeric state).

Figure S14 and S15 show the best linear fit to the calculated data (see Equation E5 and E8).

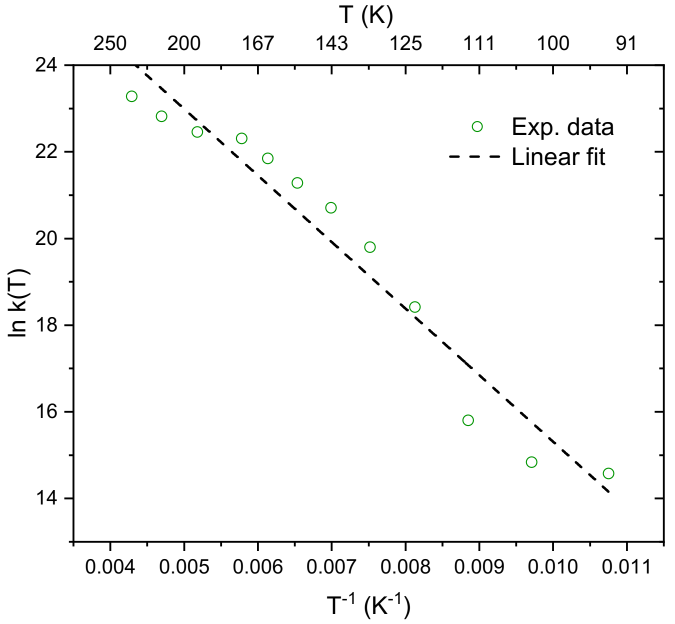

Figure S14. The natural logarithm of calculated k(T) values plotted against inverse temperature. The y-axis intersection and slope of the linear fit gives k_0 and E_a, respectively (see Equation E5).

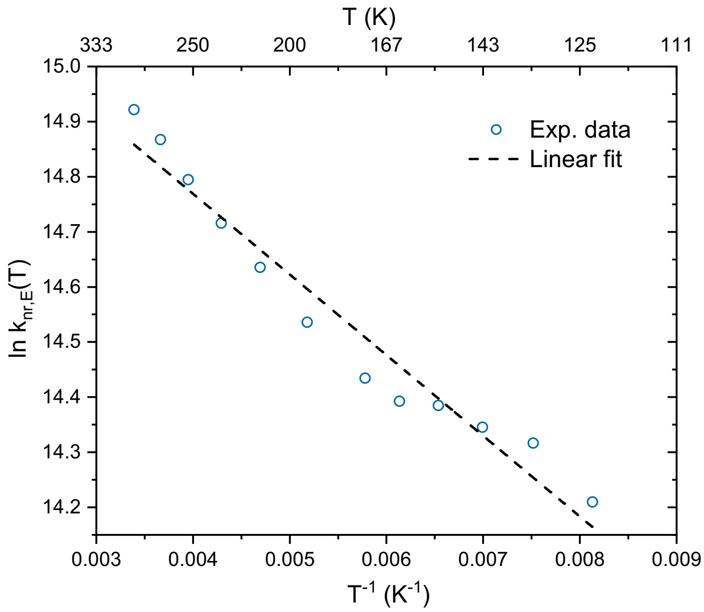

Figure S15. The natural logarithm of calculated $k_{nr,E}$(T) values plotted against inverse temperature. The y-axis intersection and slope of the linear fit gives $k_{nr,0}$ and $E_{a,nr}$, respectively (see Equation E8).

Table S1 shows the raw data that goes into the model, and the model data values that are obtained.

Table S1. Data from temperature-dependent measurements on 1,2-DPA$_2$.

Temperature (K)	Monomer lifetime (s)		Excimer lifetime (s)	
	Experimental data	Model data	Experimental data	Model data
295	-	8.74E-12	4.40E-08	4.44E-08
273	-	1.33E-11	4.43E-08	4.46E-08
253	-	2.07E-11	4.47E-08	4.48E-08
233	7.66E-11	3.48E-11	4.52E-08	4.51E-08
213	1.21E-10	6.44E-11	4.55E-08	4.54E-08
193	1.74E-10	1.35E-10	4.60E-08	4.57E-08
173	2.01E-10	3.29E-10	4.64E-08	4.61E-08
163	3.17E-10	5.50E-10	4.66E-08	4.63E-08
153	5.42E-10	9.58E-10	4.66E-08	4.66E-08
143	8.79E-10	1.71E-09	4.68E-08	4.68E-08
133	1.57E-09	2.99E-09	4.69E-08	4.71E-08
123	2.02E-09	4.72E-09	4.72E-08	4.74E-08
113	2.80E-09	6.29E-09	-	4.77E-08
103	7.34E-09	7.14E-09	-	4.81E-08
93	7.32E-09	7.44E-09	-	4.84E-08

Temperature (K)	Φ_M		Φ_E	
	Experimental data	Model data	Experimental data	Model data
295	9.41E-01	1.16E-03	9.32E-01	8.73E-01
273	1.00E-02	1.77E-03	9.17E-01	8.77E-01
253	1.09E-02	2.75E-03	9.08E-01	8.80E-01
233	1.26E-02	4.63E-03	9.01E-01	8.84E-01
213	1.33E-02	8.56E-03	8.94E-01	8.86E-01
193	1.50E-01	1.79E-02	8.90E-01	8.84E-01
173	2.11E-02	4.37E-02	8.86E-01	8.68E-01
163	2.90E-02	7.31E-02	8.69E-01	8.45E-01
153	5.22E-02	1.27E-01	8.47E-01	8.00E-01
143	1.34E-01	2.28E-01	7.74E-01	7.12E-01
133	3.76E-01	3.98E-01	5.52E-01	5.58E-01
123	7.98E-01	6.28E-01	1.70E-01	3.47E-01
113	9.80E-01	8.36E-01	0	1.54E-01
103	9.80E-01	9.50E-01	0	4.71E-02
93	9.84E-01	9.90E-01	0	9.94E-03

4. Photophysical characterization

4.1 Upconversion measurements

Figure S16 to S19 show spectra from steady-state upconversion measurements. The fittings in Figure S18-S19 were done by normalizing the fluorescence spectra from optically dilute samples of the annihilators alone to that of the UC measurements. This was done in order to estimate the generated amount of actual UC emission,[7] as especially the more blueshifted parts of the UC spectra are distorted due to secondary inner-filter effects. The normalizing was done at 470 nm where the UC samples have negligible absorbance.

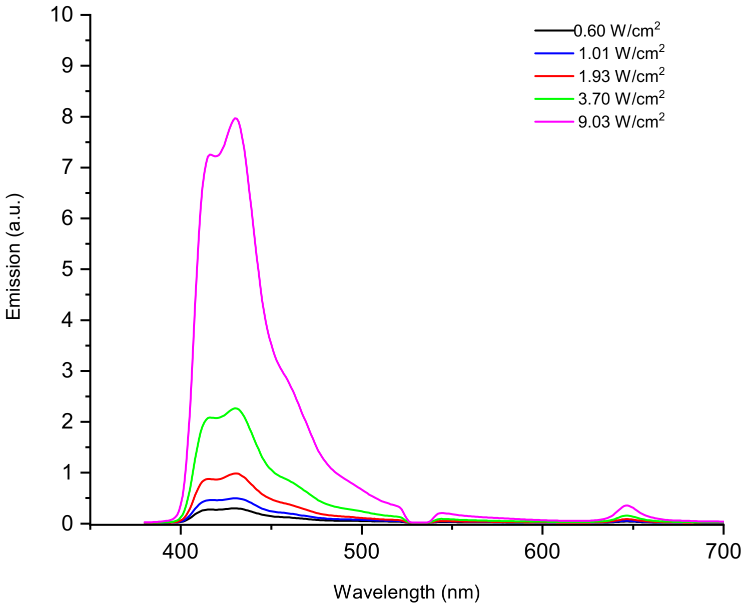

Figure S16. Upconverted emission of 1 mM DPA in degassed toluene in presence of 6.6 µM PtOEP under 532 nm cw excitation.

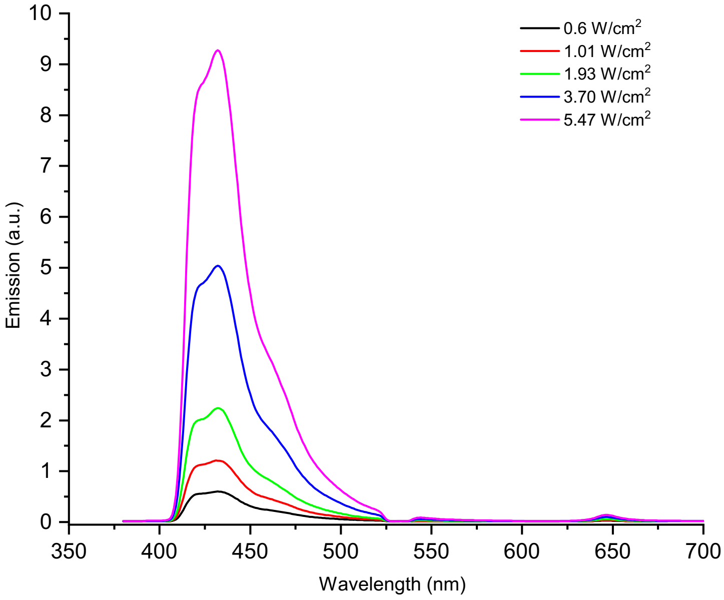

Figure S17. Upconverted emission of 0.5 mM 1,3-DPA_2 in toluene in presence of 6.6 µM PtOEP under 532 nm cw excitation.

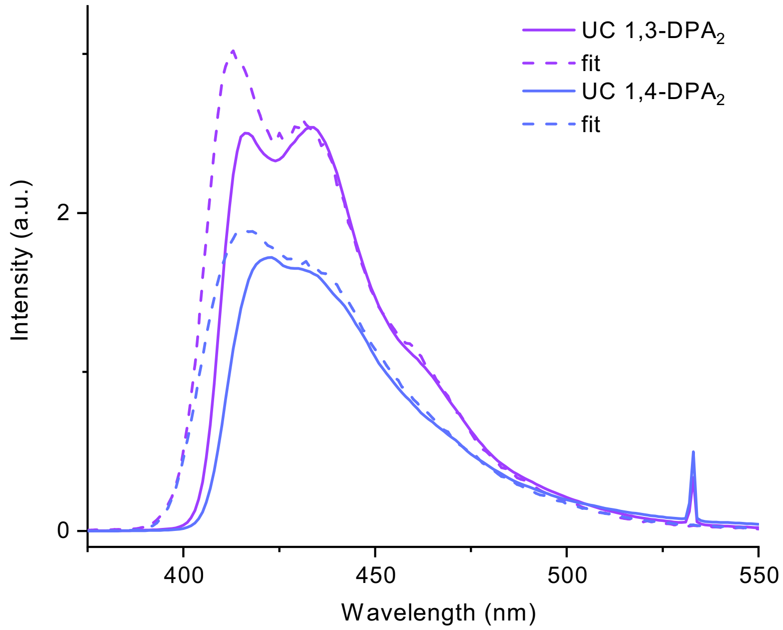

Figure S18. Fitting of UC spectra to optically dilute spectra of 1,3-DPA$_2$ and 1,4-DPA$_2$ for determination of internal UC quantum yield.

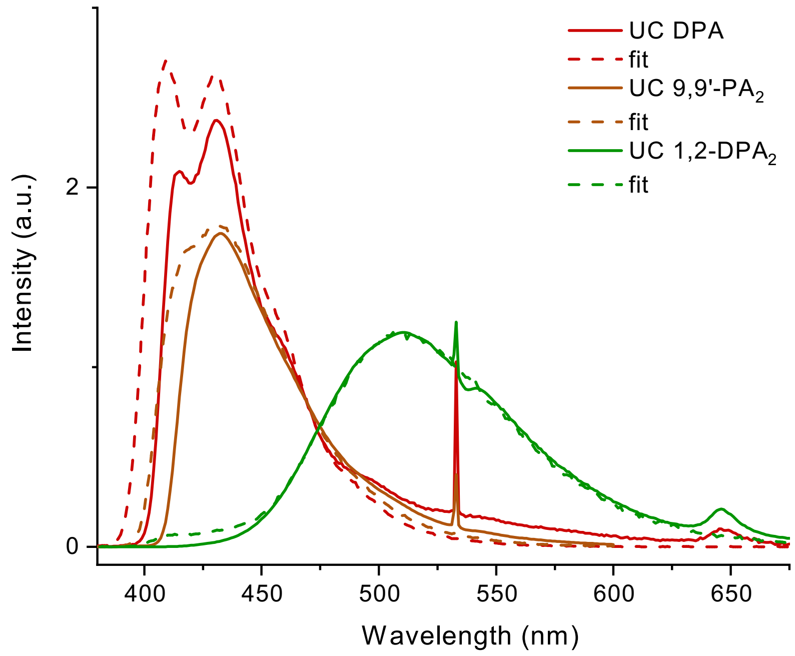

Figure S19. Fitting of UC spectra to optically dilute spectra of DPA, 9,9'-PA$_2$, and 1,2-DPA$_2$ for determination of internal UC quantum yield.

4.2 Determination of triplet excited state lifetimes

As mentioned in the main text, the triplet lifetimes of the sensitizer and annihilators were determined using time-resolved emission measurements. All compounds may undergo TTA, adding a second possible loss-channel to the kinetics of the triplet excited states. To take into account both first-order and second-order decay, Equation S1 is used for fitting.

$$I(t) \propto [\,^{3}A^{*}]^{n} = ([\,^{3}A^{*}]_{0}\frac{1-\beta}{exp(t/\tau_{T})-\beta})^{n} \quad \text{(S1)}$$

Here, I(t) is the emission intensity, β is a dimensionless parameter between 0 and 1 expressing what fraction of initial decay which is governed by second-order channels (with β = 1 meaning all initial decay is of second order), t is time, τ_T is the triplet excited state lifetime, and n is a dimensionless parameter describing the emission power dependency on the triplet concentration. The analytical expression for β is given by Equation S2:

$$\beta = \frac{2k_{TTA}[\,^{3}A^{*}]_{0}}{k_{T} + 2k_{TTA}[\,^{3}A^{*}]_{0}} \quad \text{(S2)}$$

where k_{TTA} and k_T (= $1/\tau_T$) are the rate constants for second-order TTA decay and first-order intrinsic triplet decay, respectively. In the case of PtOEP, the phosphorescence emission is used to determine τ_T. Since phosphorescence is emission directly from the triplet excited state, n = 1 during fitting. In the case of annihilators, the UC emission, which is the result of second-order TTA events, is measured. In that instance, n = 2 during fittings (and Equation S1 is then identical to Equation 5 in the main text).

The fraction of initial decay that will proceed through second-order channels is dependent on excitation intensity as this controls the initial concentration of excited triplets. Therefore, emission measurements were performed at several different intensities of the 532 nm excitation pump, and the kinetics were then globally fitted to Equation S1, using a shared τ_T but with β individually fitted to each trace. Figure S20 shows the global fit to PtOEP phosphorescence, recorded at 646 nm, at four different excitation intensities. Figure S21 to S24 shows the global fittings to the UC emission decay, recorded at 430 nm (510 nm for 1,2-DPA_2), of UC samples containing the different annihilators. The triplet lifetimes are summarized in Table 2 of the main text.

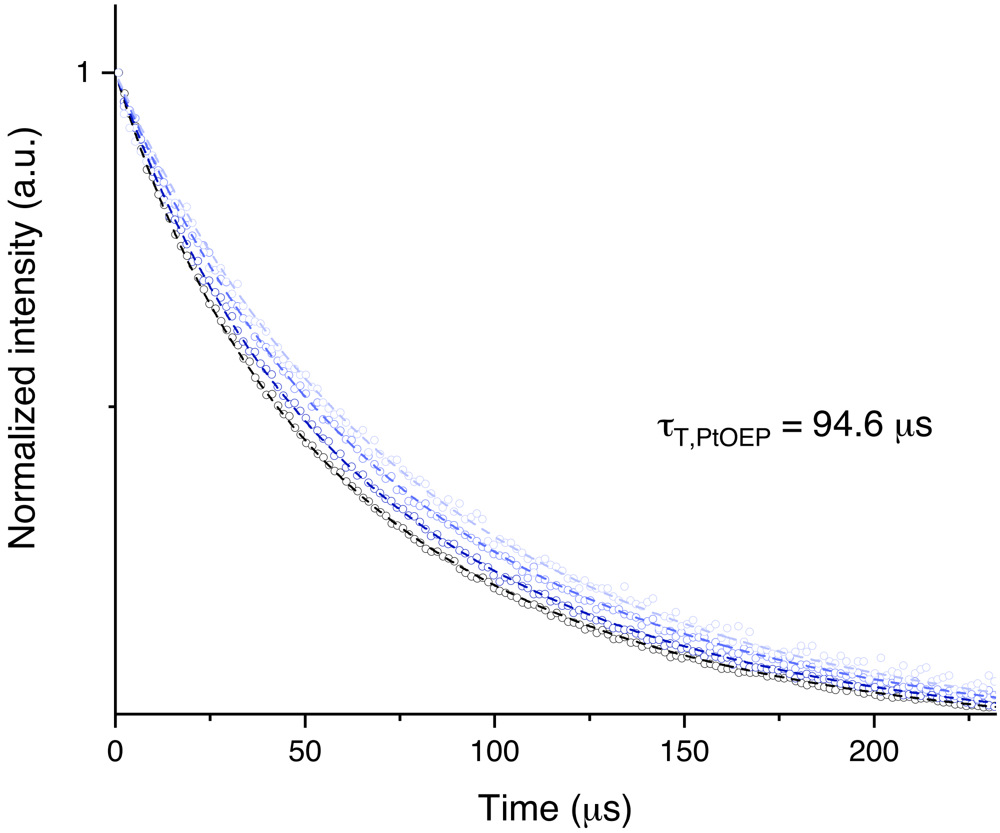

Figure S20. Global fitting of the phosphorescence (λ_{em} = 646 nm) from c. 5 μM PtOEP in deoxygenated toluene, fitted to Equation S1 with n = 1.

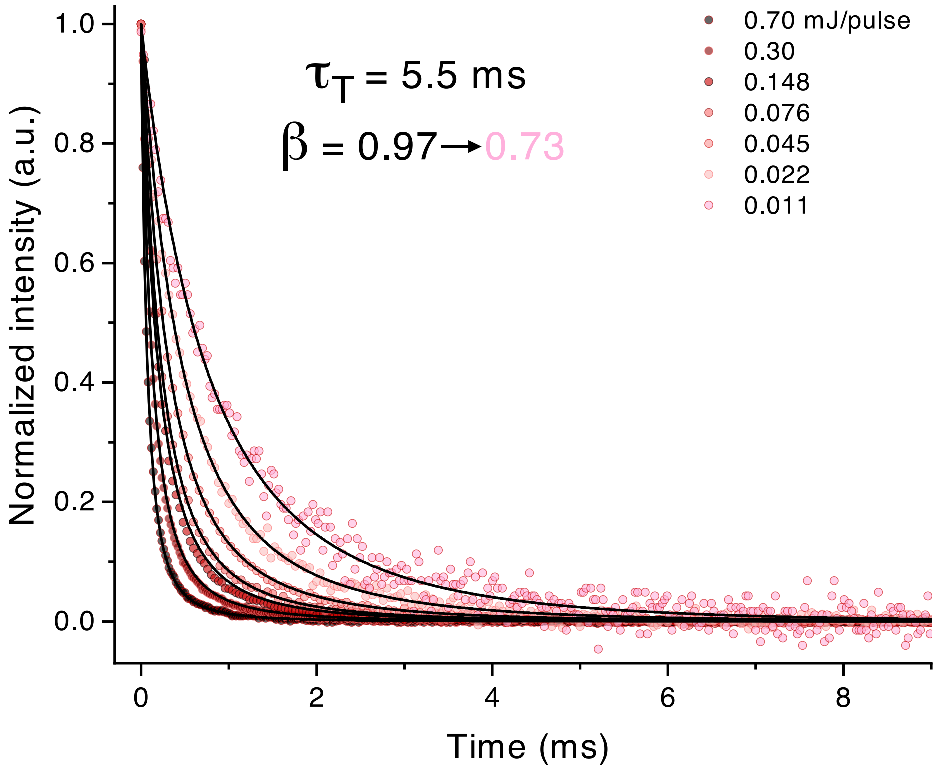

Figure S21. Global fitting of the delayed UC fluorescence (λ_{em} = 430 nm) from 6.6 μM PtOEP/1 mM DPA in deoxygenated toluene, fitted to Equation S1 with n = 2.

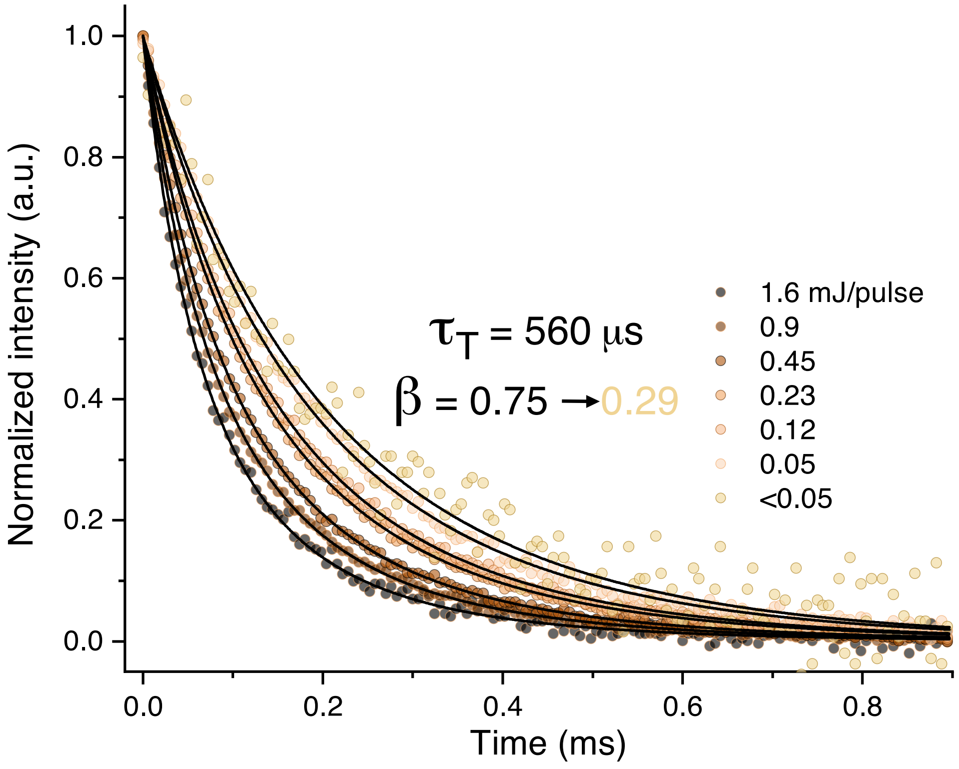

Figure S22. Global fitting of the delayed UC fluorescence (λ_{em} = 430 nm) from 6.6 μM PtOEP/0.5 mM 9,9'-PA_2 in deoxygenated toluene, fitted to Equation S1 with n = 2.

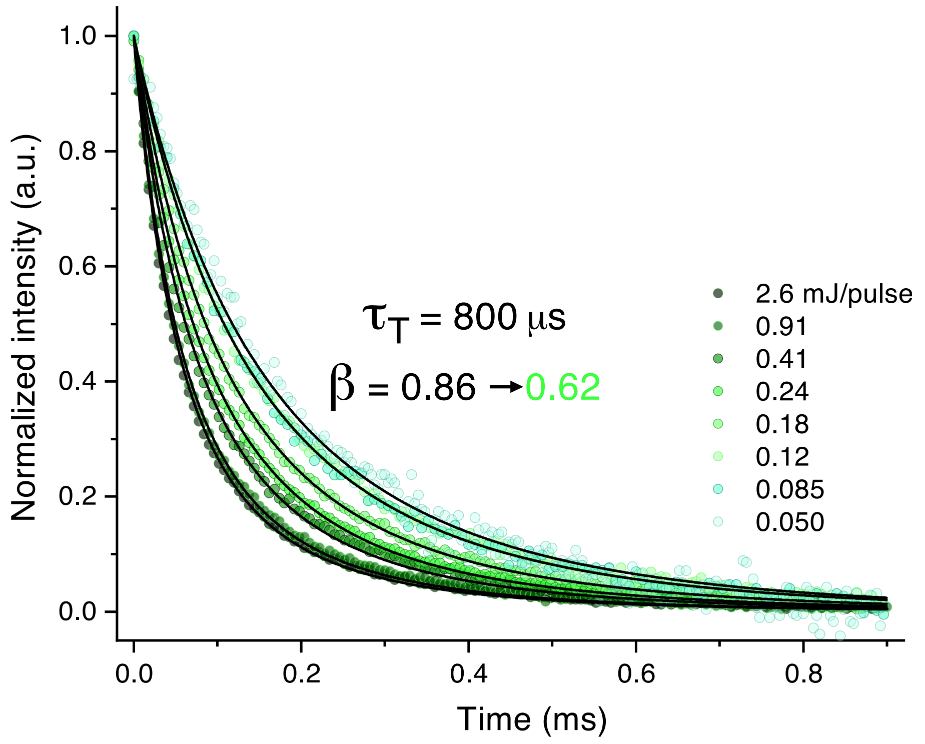

Figure S23. Global fitting of the delayed UC fluorescence (λ_{em} = 510 nm) from 6.6 μM PtOEP/0.5 mM 1,2-DPA_2 in deoxygenated toluene, fitted to Equation S1 with n = 2.

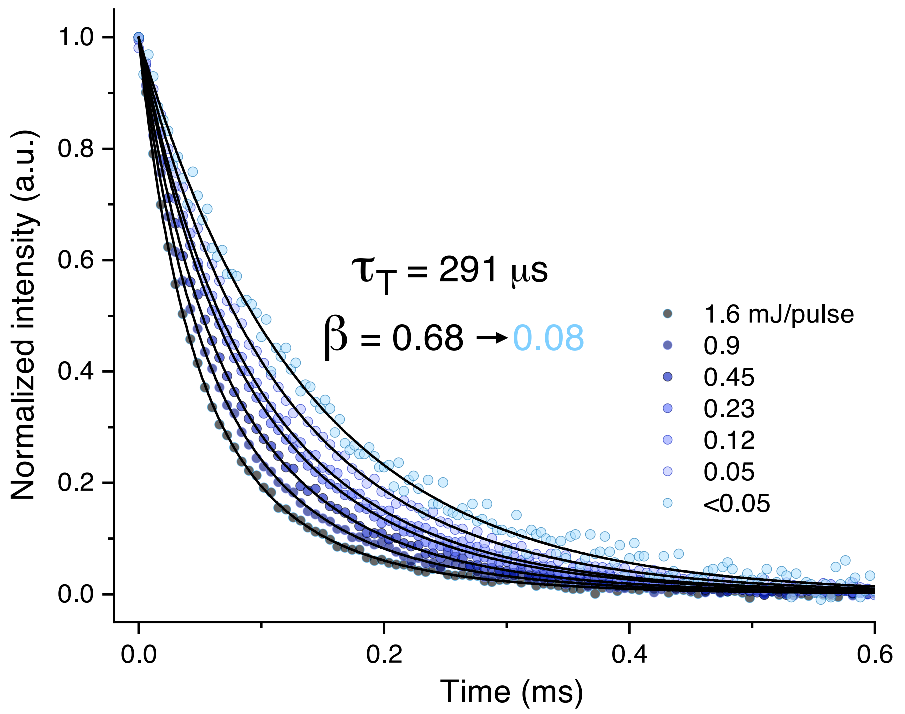

Figure S24. Global fitting of the delayed UC fluorescence (λ_{em} = 430 nm) from 6.6 μM PtOEP/0.5 mM 1,4-DPA$_2$ in deoxygenated toluene, fitted to Equation S1 with n = 2.

It is interesting to compare the results from a single-exponential tail fit with that of the fit based on Equation S1 with n=2 (from hereon called the "beta fit"). The results for high (0.70 mJ/pulse) and low (0.011 mJ/pulse) excitation intensity measurements of DPA are presented in Figure S25. Each trace has been individually fitted in contrast to the fitting procedure of Figure S20-S24. It is obvious that at high excitation intensities (leading to very high TTA efficiencies, i.e. high β) neither the tail fit or the beta fit give satisfactory results, under- and overestimating the triplet lifetime, respectively. At the lowest excitation intensity that yielded a detectable emission signal in our system, the tail fit yields a triplet lifetime approx. a factor of three smaller than that obtained from the global fit (Figure S21). The beta fit more or less mimics the results of the global fitting procedure, yielding a similar triplet lifetime of 5.8 ms. It should be noted that the single-exponential fits are particularly poor at earlier times where second-order decay of excited triplets dominates (Figure S25B and S25D).

The tail fit procedure is especially unreliable for systems with high TTA efficiencies (such as low-viscosity systems with DPA) as the triplet lifetime can be gravely underestimated if experiments aren't carefully conducted at extremely low excitation intensities. The global fitting procedure might be superfluous in systems with much lower TTA efficiencies but presents a robust and universal platform for reliable determination of triplet lifetimes for any upconverting system. In addition, this method uses the upconverted emission signals, which are typically easier to measure than the weak triplet absorption signals from transient absorption measurements that are often used. Furthermore, the obtained β values (and especially the highest one, i.e. 0.97 for DPA) are excellent indicators of TTA efficiency, which are useful when evaluating the overall upconversion performance of a system.

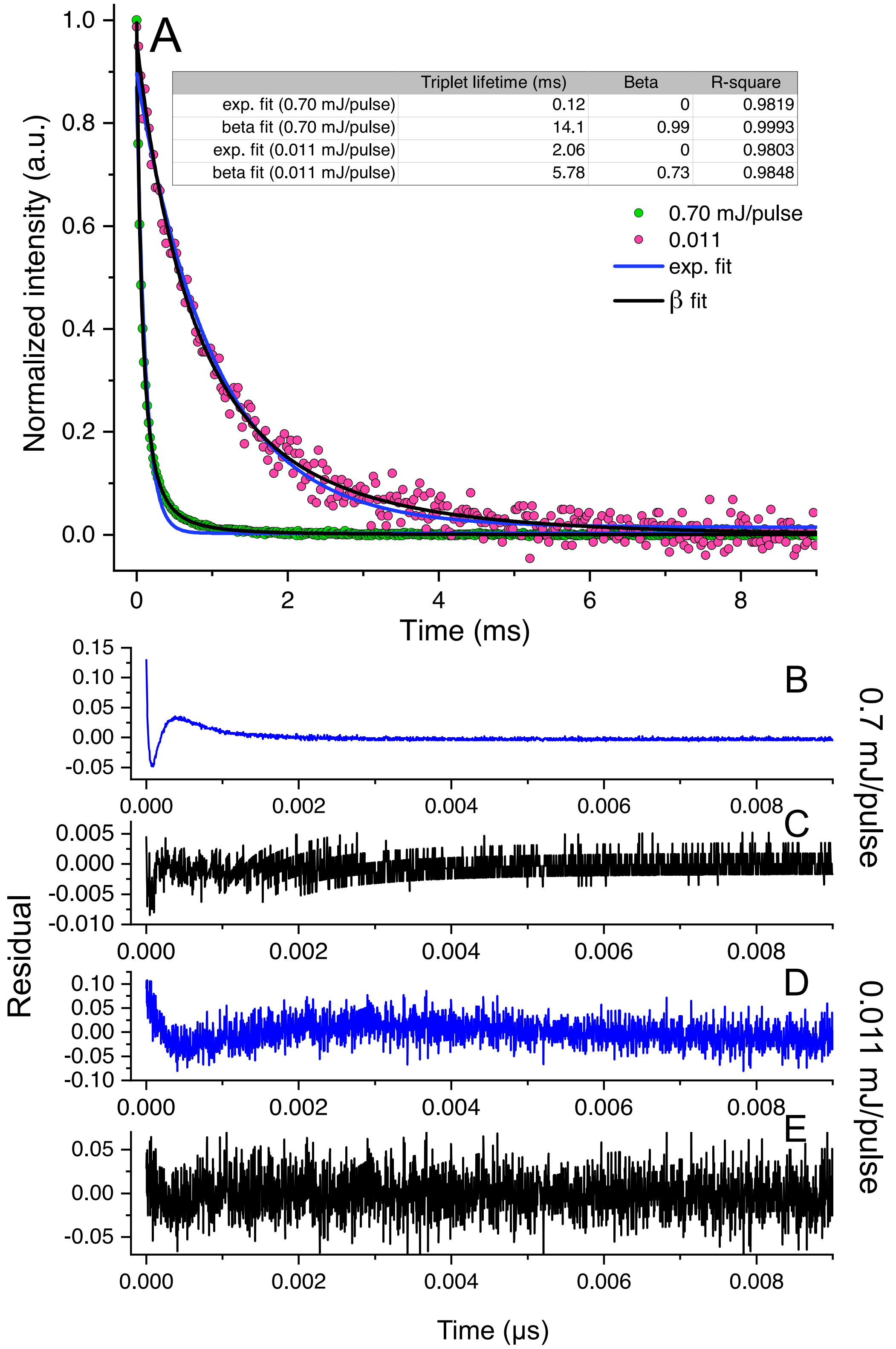

Figure S25. (A) Comparison between a single-exponential fit and fitting according to Equation S1 with n=2, for the DPA measurements with highest and lowest excitation intensities, respectively. Here, each trace is individually fitted. (B-E) Fitting residuals for (B) single-exponential fitting and (C) beta fitting at 0.70 mJ/pulse excitation intensity, and for (D) single-exponential fitting and (E) beta fitting at 0.011 mJ/pulse excitation intensities.

4.3 Determination of the triplet-triplet annihilation rate constant

To determine the rate of TTA, i.e. the k_{TTA} rate constant, a procedure previously used by our group was employed.[8,9] Transient absorption measurements at two different wavelengths were performed. Assuming that sensitizer intersystem crossing is instantaneous on the timescale of the experiment, at 430 nm there are three different contributions to the observed kinetics. At early times a positive feature is observed, which is due to annihilator triplet absorption ($\varepsilon_{A,T}$) and sensitizer triplet absorption ($\varepsilon_{S,T}$). At later times this trace grows into a negative feature as delayed fluorescence from the annihilator (α_{FL}) is observed. The trace should thus be fitted to Equation 1:

$$\Delta A_{430\,nm} = \varepsilon_{A,T} \times [^3A^*] + \varepsilon_{S,T} \times [^3S^*] - \alpha_{FL} \times [^1A^*] \quad (1)$$

At 646 nm only the sensitizer phosphorescence (α_{PH}) is observed:

$$\Delta A_{646\,nm} = -\,\alpha_{PH} \times [^3S^*] \quad (2)$$

The concentrations at each time are obtained by solving the system of differential equations governing the TTA process:

$$\frac{d[^3S^*]}{dt} = -k_{PS}[\,^3S^*] - k_{TET}[\,^3S^*][^1A] \quad (3)$$

$$\frac{d[^3A^*]}{dt} = k_{TET}[\,^3S^*][^1A] - k_T[\,^3A^*] - 2k_{TTA}[\,^3A^*]^2 \quad (4)$$

$$\frac{d[^1A^*]}{dt} = k_{TTA}[\,^3A^*]^2 - k_{FA}[\,^1A^*] \quad (5)$$

$$\frac{d[^1A]}{dt} = -k_{TET}[\,^3S^*][^1A] + k_T[\,^3A^*] + k_{TTA}[\,^3A^*]^2 + k_{FA}[\,^1A^*] \quad (6)$$

Here, sensitizer TTA is assumed to be negligible compared to TET. The initial sensitizer triplet concentration is estimated from the ground state bleach at 540 nm. To cover all relevant kinetics the signals are recorded at two different time scales: the 10 μs window covers the initial absorption and the initiation of delayed fluorescence as well as sensitizer phosphorescence decay, while the 1 ms window covers the decay of delayed fluorescence following annihilator TTA. Concentrations are given by solving equations 3 to 6 using the differential equation solver ode23s in MatLAB, and the three traces (430 nm and 646 nm in 10 μs window, 430 nm in 1 ms window) are then fitted globally to equations 1 and 2 by minimizing the difference between recorded data and the fitting using the fmincon function. The parameters that are fitted are k_{TTA} and k_{TET}. $\varepsilon_{A,T}$, $\varepsilon_{S,T}$, α_{FL}, and α_{PH} are produced using a least-square method, while all remaining rate constants are taken from previous experiments and thus predefined in the program.

The results from the fitting procedure are given in Figure S26 to S30. The obtained values of k_{TET} are in reasonable agreement with those measured using Stern-Volmer quenching, see Table 2 in the main text. The emission intensity at 430 nm for 1,2-DPA_2 is very weak, and thus the fitting for this particular data set is slightly worse. The obtained rate constants are however reasonable and deemed trustworthy.

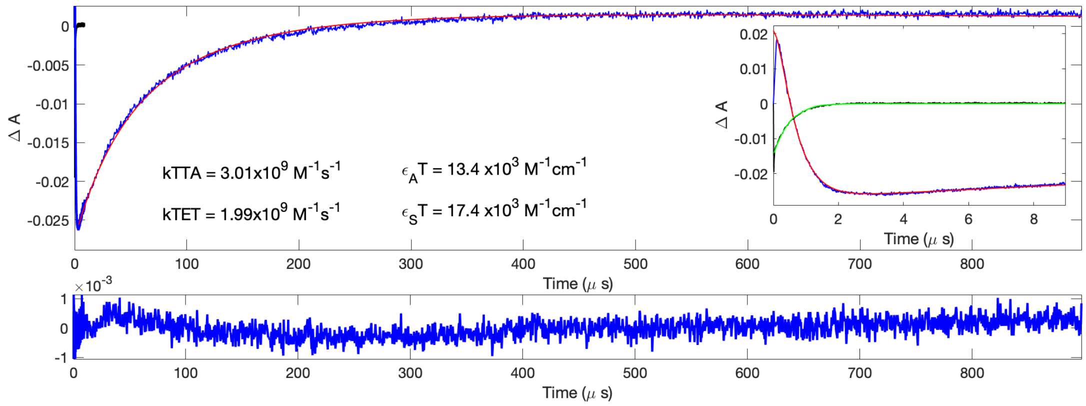

Figure S26. Transient absorption measurements at 430 nm (blue) and 646 nm (black) for 1 mM DPA and 5.8 μM PtOEP at 1 mJ/pulse pump power. Red and green traces are best fits and the bottom panel shows the residual.

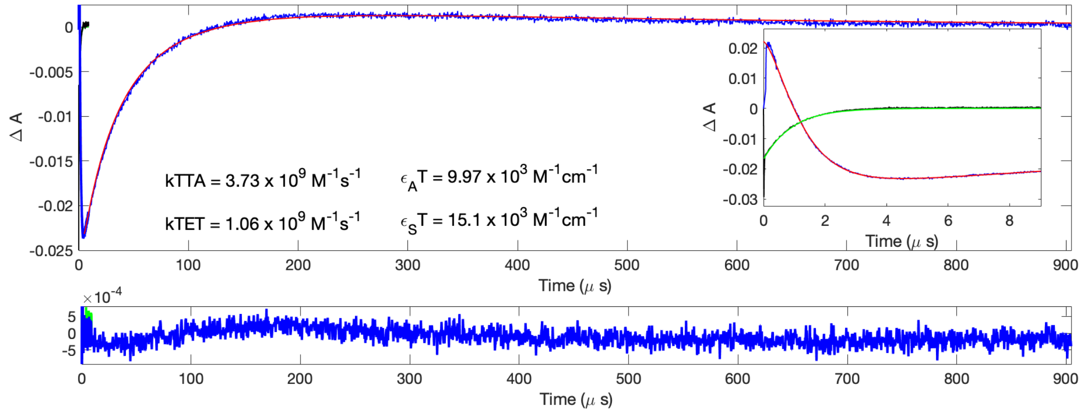

Figure S27. Transient absorption measurements at 430 nm (blue) and 646 nm (black) for 0.5 mM 9,9’-PA$_2$ and 5.8 μM PtOEP at 1 mJ/pulse pump power. Red and green traces are best fits and the bottom panel shows the residual.

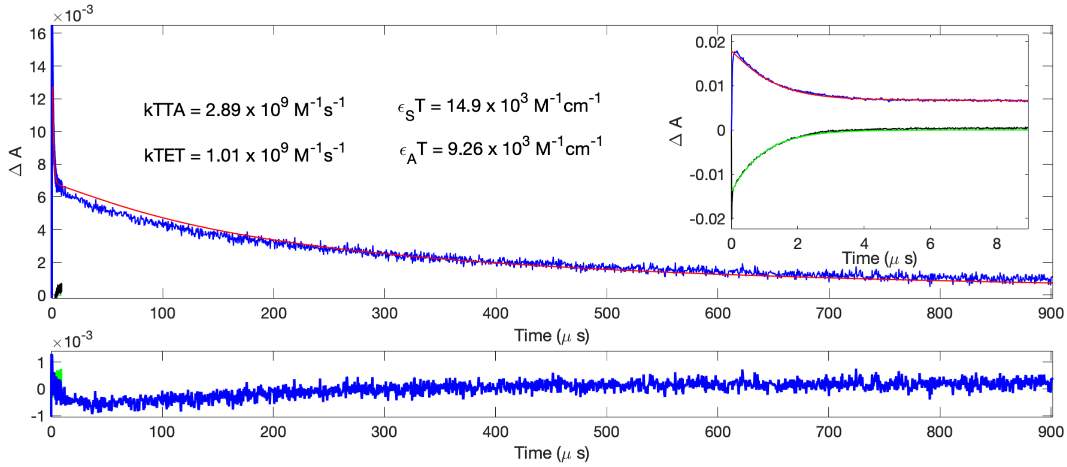

Figure S28. Transient absorption measurements at 430 nm (blue) and 646 nm (black) for 0.5 mM 1,2-DPA$_2$ and 5.8 μM PtOEP at 1 mJ/pulse pump power. Red and green traces are best fits and the bottom panel shows the residual.

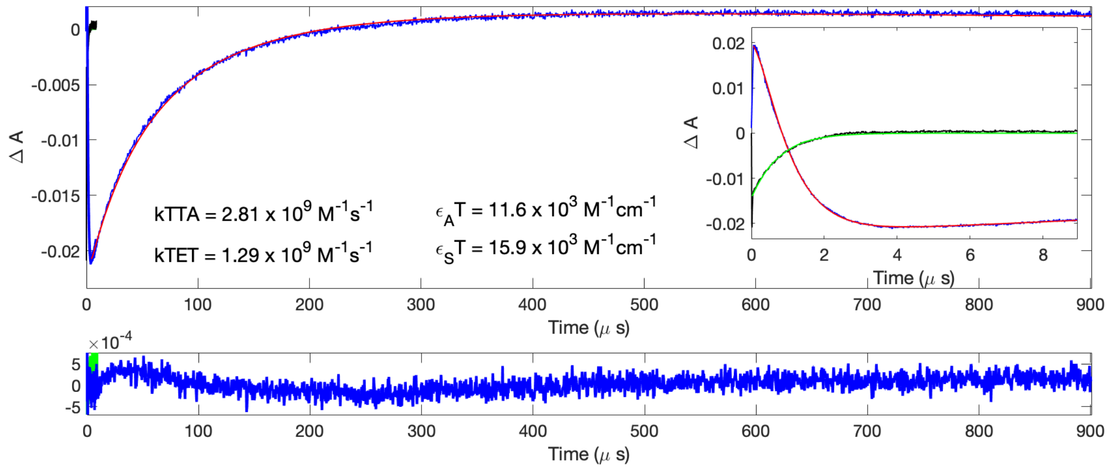

Figure S29. Transient absorption measurements at 430 nm (blue) and 650 nm (black) for 0.5 mM 1,3-DPA$_2$ and 5.8 μM PtOEP at 1 mJ/pulse pump power. Red and green traces are best fits and the bottom panel shows the residual.

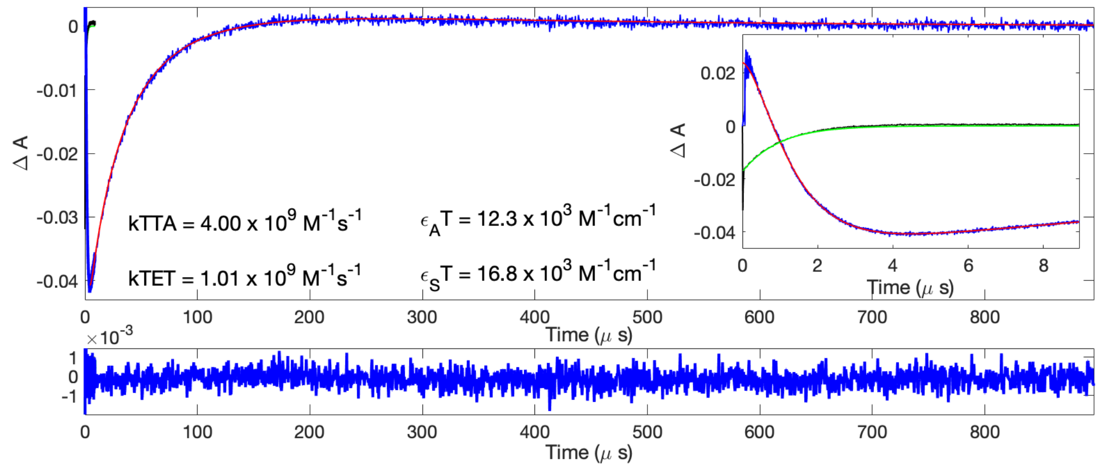

Figure S30. Transient absorption measurements at 430 nm (blue) and 650 nm (black) for 0.5 mM 1,4-DPA$_2$ and 5.8 μM PtOEP at 1 mJ/pulse pump power. Red and green traces are best fits and the bottom panel shows the residual.

Table S2. Best fit parameters to the transient absorption measurements.

	**k_{TTA} (×10^9 M^{-1} s^{-1})**	**k_{TET} (×10^9 M^{-1} s^{-1})^a**	**$\Delta\varepsilon_{A,T}$ (× 10^3 M^{-1} cm^{-1})**	**$\Delta\varepsilon_{S,T}$ (× 10^3 M^{-1} cm^{-1})**
**DPA**	3.01	1.99	13.4	17.4
**9,9'-PA$_2$**	3.73	1.06	9.97	15.1
**1,2-DPA$_2$**	2.89	1.01	9.26	14.9
**1,3-DPA$_2$**	2.81	1.29	11.5	15.9
**1,4-DPA$_2$**	4.00	1.01	12.3	16.8

^{a}In DPA subunits concentration.

4.4 Calculations on electronic coupling of triplet excited states

The electronic coupling for triplet energy transfer, V_{TT}, between the two DPA moieties in a dimer could be used as a proxy for how perturbed the lowest triplet excited state is compare to the parent (isolated) DPA chromophore. It should also be related to the rate constant for intramolecular TTA, but the relation is unknown at this point. Here, we use a simple TDDFT calculation of V_{TT} to compare the ortho-, meta-, and para-coupled dimers in terms of their ability to do TTA-UC and specifically to compare the quite different lowest triplet excited lifetimes of these dimers.

Calculation of V_{TT} can be done in several ways. The most direct is to use the Landau-Zener approximation which relates the electronic coupling between two states at the avoided crossing geometry to the splitting between these two states.[10] For a dimeric compound possessing a symmetry element the avoided crossing geometry is automatically fulfilled. Scheme S1 shows schematically how the electronic coupling is related to the splitting between the two nearly degenerate lowest triplet states in a dimer. TDDFT calculations (B3LYP/6-31G(2d,p)) were done with constraints to C_2-symmetry and results for the lowest excited states are given in Table S3. The calculated results are close to identical with the ones for symmetry unrestricted dimers (cf. Table 2). As expected, the energy splitting is small in all three dimers and it is smallest for the meta-coupled dimer indicating that this is the dimer with best preserved DPA properties, such as the triplet lifetime. There is however not a quantitative correspondence between the coupling and the triplet lifetime, i.e. despite 1,4-DPA_2 having a rather weak coupling it possesses the shortest triplet lifetime of the dimers (Figure S24).

Scheme S1. Relation between the energy splitting of the two lowest, nearly degenerate, triplet excited state of a dimeric DPA compound to the electronic coupling for triplet energy transfer. The calculation of the excited state energies (vertical transitions from the ground state) should be done at the avoided crossing geometry, here accomplished by optimizing while restricting the molecules to belong to the C_2 point group.

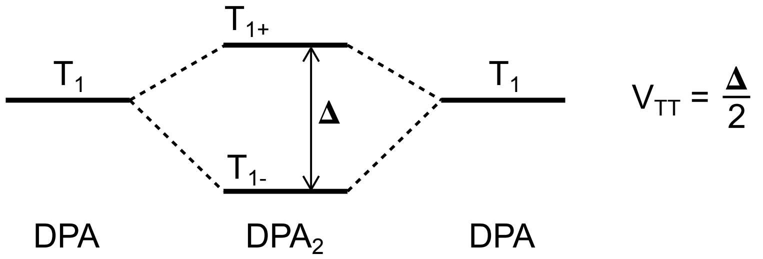

Table S3. Calculated (TDDFT, B3LYP/6-31G(2d,p)) vertical excitation energies and splitting between the two lowest triplet excited states for C_2-symmetry restricted dimers and DPA.

	S_1 (eV)	T_1 (eV)	Δ (meV)
**DPA**	3.05	1.72	---
**1,2-DPA_2**	2.85	1.71	8.1
**1,3-DPA_2**	3.06	1.74	2.0
**1,4-DPA_2**	3.06	1.72	2.4

5. Intra-molecular upconversion: Rate equations, simulations, and measurements

Steady-state and time-resolved simulations were performed using MatLAB® 2018b (MathWorks®).

5.1 Model descriptions

Our model is based on the following reactions:

$^{1}S \xrightarrow{k_{exc}} {}^{1}S^{*}$	Excitation	(R1)
$^{1}S^{*} \xrightarrow{k_{ISC}} {}^{3}S^{*}$	Intersystem crossing (ISC)	(R2)
$^{3}S^{*} + {}^{3}S^{*} \xrightarrow{k_{TTAS}} {}^{1}S + {}^{1}S^{*}$	TTA of sensitizer	(R3)
$^{3}S^{*} \xrightarrow{k_{PS}} {}^{1}S$	Excited triplet decay (phosphorescence) of sensitizer	(R4)
$^{3}S^{*} + {}^{1}A \xrightarrow{k_{TET1}} {}^{1}S + {}^{3}A^{*}$	Primary triplet energy transfer (TET) from triplet excited sensitizer to ground-state annihilator	(R5)
$^{3}A^{*} + {}^{3}A^{*} \xrightarrow{k_{TTA1}} {}^{1}A + {}^{1}A^{*}$	*inter*-molecular triplet-triplet annihilation (TTA)	(R6)
$^{3}A^{*} \xrightarrow{k_{T}} {}^{1}A$	Excited triplet decay of annihilator	(R7)
$^{1}A^{*} \xrightarrow{k_{FA}} {}^{1}A$	Excited singlet decay (fluorescence) of annihilator	(R8)

For systems which allow for *intra*-molecular TTA to proceed (i.e. where the annihilator is dimeric in nature), the following reactions are also included:

TETA model:

$$^{3}A^{*} - {}^{1}A + {}^{3}A^{*} - {}^{1}A \xrightarrow{k_{TET2}} {}^{3}A^{*} - {}^{3}A^{*} + {}^{1}A - {}^{1}A$$

Secondary triplet energy transfer (TET) from triplet excited annihilator to triplet excited annihilator (R9a)

DS model:

$$^{3}S^{*} + {}^{3}A^{*} - {}^{1}A \xrightarrow{k_{TET2}} {}^{1}S + {}^{3}A^{*} - {}^{3}A^{*}$$

Secondary triplet energy transfer (TET) from triplet excited sensitizer to triplet excited annihilator (R9b)

The *intra*-molecular TTA step is the same for both models:

$^{3}A^{*} - {}^{3}A^{*} \xrightarrow{k_{TTA2}} {}^{1}A^{*} - {}^{1}A$ *intra*-molecular triplet-triplet annihilation (TTA) (R10)

The product of reaction R10 ($^1A^*$-^1A) is from a modelling perspective interpreted as $^1A^*$, thus ultimately decaying according to reaction R8.

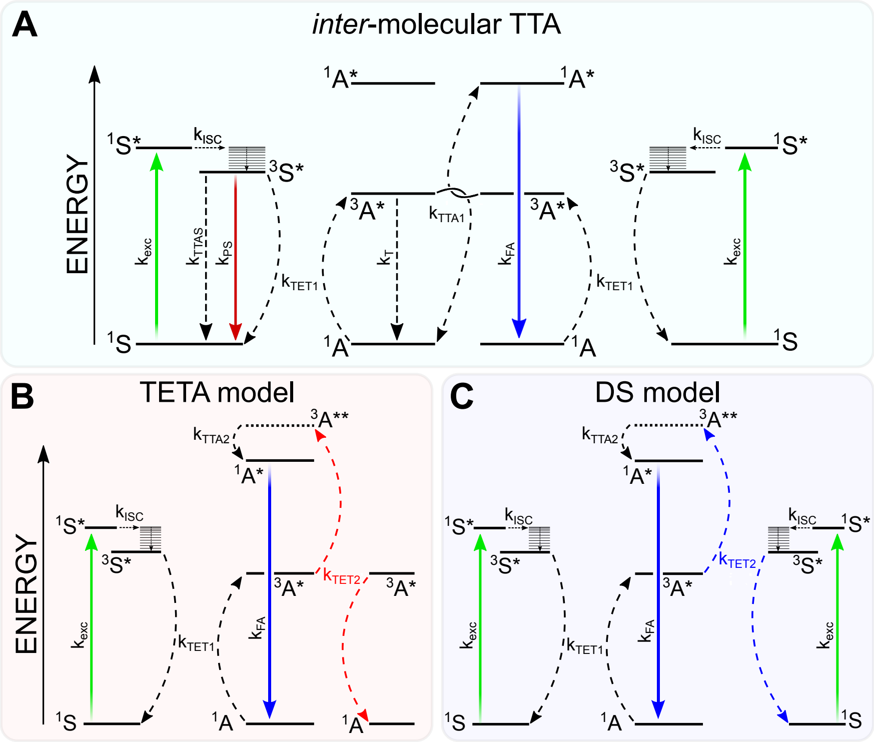

Figure S31. Jablonski plots illustrating the different processes taking place in the upconverting systems according to models of (A) *inter*-molecular TTA, (B) TETA, and (C) DS. The processes depicted by rate constants k_{TTAS}, k_{PS}, k_T, and k_{TTA1} in panel (A) are also included in the TETA and DS models, but omitted in panels (B) and (C) for clarity.

5.2 Time-resolved simulations

The time-resolved simulations are based on the equations presented below. **Bold terms** are distinct to the specified model only. For the DPA monomer all terms including either k_{TTA2}, k_{TET2}, or $[^1A^*]_{intra}$ are omitted. $^3A^{**}$ is in this context synonymous with $^3A^*$-$^3A^*$, while $^1A^*$ equals $^1A^*$-^1A in those instances where dimeric annihilators operate.

$$DS\colon \frac{d[^1S]}{dt} = k_{TTAS}[\,^3S^*]^2 + k_{PS}[\,^3S^*] + k_{TET1}[\,^3S^*][^1A] + \boldsymbol{k_{TET2}[\,^3S^*][\,^3A^*]} - k_{exc}[^1S] \qquad \text{(V1a)}$$

$$TETA\colon \frac{d[^1S]}{dt} = k_{TTAS}[\,^3S^*]^2 + k_{PS}[\,^3S^*] + k_{TET1}[\,^3S^*][^1A] - k_{exc}[^1S] \qquad \text{(V1b)}$$

$$\frac{d[^1S^*]}{dt} = k_{TTAS}[\,^3S^*]^2 + k_{exc}[^1S] - k_{ISC}[^1S^*] \qquad \text{(V2)}$$

$$DS\colon \frac{d[^3S^*]}{dt} = k_{ISC}[^1S^*] - k_{PS}[\,^3S^*] - 2k_{TTAS}[\,^3S^*]^2 - k_{TET1}[\,^3S^*][^1A] - \boldsymbol{k_{TET2}[\,^3S^*][\,^3A^*]} \qquad \text{(V3a)}$$

$$TETA\colon \frac{d[^3S^*]}{dt} = k_{ISC}[^1S^*] - k_{PS}[\,^3S^*] - 2k_{TTAS}[\,^3S^*]^2 - k_{TET1}[\,^3S^*][^1A] \qquad \text{(V3b)}$$

$$DS\colon \frac{d[^3A^*]}{dt} = k_{TET1}[\,^3S^*][^1A] - k_T[\,^3A^*] - 2k_{TTA1}[\,^3A^*]^2 - \boldsymbol{k_{TET2}[\,^3S^*][\,^3A^*]} \qquad \text{(V4a)}$$

$$TETA\text{: } \frac{d[{}^3A^*]}{dt} = k_{TET1}[\,{}^3S^*][{}^1A] - k_T[\,{}^3A^*] - \frac{1}{4} * 2k_{TTA1}[\,{}^3A^*]^2 - \boldsymbol{k_{TET2}}[\,{}^3\boldsymbol{A}^*]^2 \quad \text{(V4b)}$$

$$DS\text{: } \frac{d[{}^3A^{**}]}{dt} = \boldsymbol{k_{TET2}}[\,{}^3\boldsymbol{S}^*][\,{}^3\boldsymbol{A}^*] - k_{TTA2}[\,{}^3A^{**}] \quad \text{(V5a)}$$

$$TETA\text{: } \frac{d[{}^3A^{**}]}{dt} = \frac{1}{2}\boldsymbol{k_{TET2}}[\,{}^3\boldsymbol{A}^*]^2 - k_{TTA2}[\,{}^3A^{**}] \quad \text{(V5b)}$$

$$DS\text{: } \frac{d[{}^1A^*]_{inter}}{dt} = k_{TTA1}[\,{}^3A^*]^2 - k_{FA}[\,{}^1A^*]_{inter} \quad \text{(V6a)}$$

$$TETA\text{: } \frac{d[{}^1A^*]_{inter}}{dt} = \frac{1}{4}k_{TTA1}[\,{}^3A^*]^2 - k_{FA}[\,{}^1A^*]_{inter} \quad \text{(V6b)}$$

$$\frac{d[{}^1A^*]_{intra}}{dt} = k_{TTA2}[\,{}^3A^{**}] - k_{FA}[\,{}^1A^*]_{intra} \quad \text{(V7)}$$

$$DS\text{: } \frac{d[{}^1A]}{dt} = k_{TTA1}[\,{}^3A^*]^2 + k_{FA}([\,{}^1A^*]_{inter} + [\,{}^1A^*]_{intra}) + k_T[\,{}^3A^*] - k_{TET1}[\,{}^3S^*][{}^1A] \quad \text{(V8a)}$$

$$TETA\text{: } \frac{d[{}^1A]}{dt} = \frac{1}{4}k_{TTA1}[\,{}^3A^*]^2 + \frac{1}{2}\boldsymbol{k_{TET2}}[\,{}^3\boldsymbol{A}^*]^2 + \boldsymbol{k_{FA}}([\,{}^1A^*]_{inter} + [\,{}^1A^*]_{intra}) + k_T[\,{}^3A^*] - k_{TET1}[\,{}^3S^*][{}^1A] \quad \text{(V8b)}$$

Differential equations V1-V8 were solved using MatLAB's ODE solver ode23tb. The total concentration of excited singlet annihilators is found simply by addition of the *inter*- and *intra*-molecular TTA products (equation V6 and V7).

The results from the time-resolved simulations are presented in Figure S32. Two different sensitizer (platinum octaethylporphyrin, PtOEP) concentrations are investigated: 5 μM (typical upconversion sample) and 100 μM. The TETA model predicts a slower decay of the UC emission than the DS model, while the DS model predicts a faster rise time than the TETA model. These tendencies are further accentuated when increasing the sensitizer concentration or lowering the annihilator concentration (right panel of Figure S32).

5.3 Steady-state simulations

The steady-state simulations are based on the equations presented below. **Bold terms** are distinct to the specified model only. For the DPA monomer all terms including either k_{TTA2}, k_{TET2}, or $[{}^1A^*]_{intra}$ are omitted. ${}^3A^{**}$ is in this context synonymous with ${}^3A^*$-${}^3A^*$, while ${}^1A^*$ equals ${}^1A^*$-${}^1A$ in those instances where dimeric annihilators operate.

$$0 = [{}^1S] + [{}^1S*] + [{}^3S*] - [{}^1S]_0 \quad \text{(T1)}$$

$$0 = k_{TTAS}[\,{}^3S^*]^2 + k_{exc}[{}^1S] - k_{ISC}[{}^1S^*] \quad \text{(T2)}$$

$$DS\text{: } 0 = k_{ISC}[{}^1S^*] - k_{PS}[\,{}^3S^*] - 2k_{TTAS}[\,{}^3S^*]^2 - k_{TET1}[\,{}^3S^*][{}^1A] - \boldsymbol{k_{TET2}}[\,{}^3\boldsymbol{S}^*][\,{}^3\boldsymbol{A}^*] \quad \text{(T3a)}$$

$$TETA\text{: } 0 = k_{ISC}[{}^1S^*] - k_{PS}[\,{}^3S^*] - 2k_{TTAS}[\,{}^3S^*]^2 - k_{TET1}[\,{}^3S^*][{}^1A] \quad \text{(T3b)}$$

$$DS\text{: } 0 = k_{TET1}[\,{}^3S^*][{}^1A] - k_T[\,{}^3A^*] - 2k_{TTA1}[\,{}^3A^*]^2 - \boldsymbol{k_{TET2}}[\,{}^3\boldsymbol{S}^*][\,{}^3\boldsymbol{A}^*] \quad \text{(T4a)}$$

$$TETA\text{: } 0 = k_{TET1}[\,{}^3S^*][{}^1A] - k_T[\,{}^3A^*] - \frac{1}{4} * 2k_{TTA1}[\,{}^3A^*]^2 - \boldsymbol{k_{TET2}}[\,{}^3\boldsymbol{A}^*]^2 \quad \text{(T4b)}$$

$$DS: 0 = \boldsymbol{k_{TET2}}[\,^3\boldsymbol{S}^*][\,^3\boldsymbol{A}^*] - k_{TTA2}[\,^3A^{**}] \quad \text{(T5a)}$$

$$TETA: 0 = \frac{1}{2}\boldsymbol{k_{TET2}}[\,^3\boldsymbol{A}^*]^2 - k_{TTA2}[\,^3A^{**}] \quad \text{(T5b)}$$

$$DS: 0 = k_{TTA1}[\,^3A^*]^2 - k_{FA}[\,^1A^*]_{inter} \quad \text{(T6a)}$$

$$TETA: 0 = \frac{1}{4}k_{TTA1}[\,^3A^*]^2 - k_{FA}[\,^1A^*]_{inter} \quad \text{(T6b)}$$

$$0 = k_{TTA2}[\,^3A^{**}] - k_{FA}[\,^1A^*]_{intra} \quad \text{(T7)}$$

$$0 = [\,^3A^{**}] + [\,^3A^*] + [\,^1A^*] + [^1A] - [^1A]_0 \quad \text{(T8)}$$

The material balances T1-T8 were solved using MatLAB's nonlinear equations solver fsolve. The steady-state total concentration of excited singlet annihilators is found simply by addition of the *inter*- and *intra*-molecular TTA products. Figure S33 to S36 show the results of a few simulations performed, highlighting what behavior to be expected from our systems depending on which *intra*-molecular upconversion mechanism is present. The simulated excited singlet annihilator concentration multiplied by the singlet excited state lifetime (the product is assumed to be proportional to UC emission intensity) is then plotted against the initial ground state annihilator concentration, $[A]_0$. It should be noted that the spin-statistical factor is not included in the models, and that eventual inner-filter effects are not simulated as the inclusion of such should not cause any differences when comparing the different models with each other.

5.4 Settings and parameters

The parameters and rate constants used during simulations are given in Table S4. These values were used consistently for the simulations in Figures S32 to S37. Remaining parameters used (k_{TET}, k_{TTA}, k_{FA} (= $1/\tau_F$), k_T) can be found in Table 1 and Table 2 in the main text.

Table S4. Parameters and rate constants used during steady-state and time-resolved simulations.

	**Power density (W/cm^2 (SS) or mJ/pulse (TR))**	**Excitation wavelength (nm)**	**Excitation path length (cm)**	**Laser pulse diameter (cm)**	**Sample volume (ml)**	**k_{TTAS} (M^{-1} s^{-1})**	**k_{PS} (s^{-1})**
Steady-state	13[a]	532	1	0.07[c]	0.7[e]	10^9	1.09*10^4
Time-resolved	1[b]	532	1	1[d]	1.4[f]	10^9	1.09*10^4

	**k_{exc} (s^{-1})**	**k_{ISC} (s^{-1})**	**k_{TET2}[h] (M^{-1} s^{-1})**	**k_{TTA2} (M^{-1} s^{-1})**
Steady-state	5100[g]	10^{10}	k_{TET1}	10^{12}
Time-resolved		10^{10}	k_{TET1}	10^{12}

[a]Power density used for experimental determination of ϕ_{UC}. [b]Typical pump power during ns-TA measurements. [c]Spot diameter of 532 nm OBIS laser. [d]Estimated pump diameter during ns-TA. [e]Steady-state measurements performed in 2×10 mm cuvette, long side parallel to pump direction. [f]ns-TA measurements performed in 4×10 mm cuvette. [g]Calculated as $k_{exc} = P_{exc} \times \alpha(532\ nm)_{PtOEP}$, where P_{exc} is the photon flux in photons cm^{-2} s^{-1}, and $\alpha(532\ nm)_{PtOEP}$ is the absorption cross-section of PtOEP in cm^2. [h]Secondary TET step rate assumed to be equal to primary TET step rate.

5.5 Simulation and experimental results

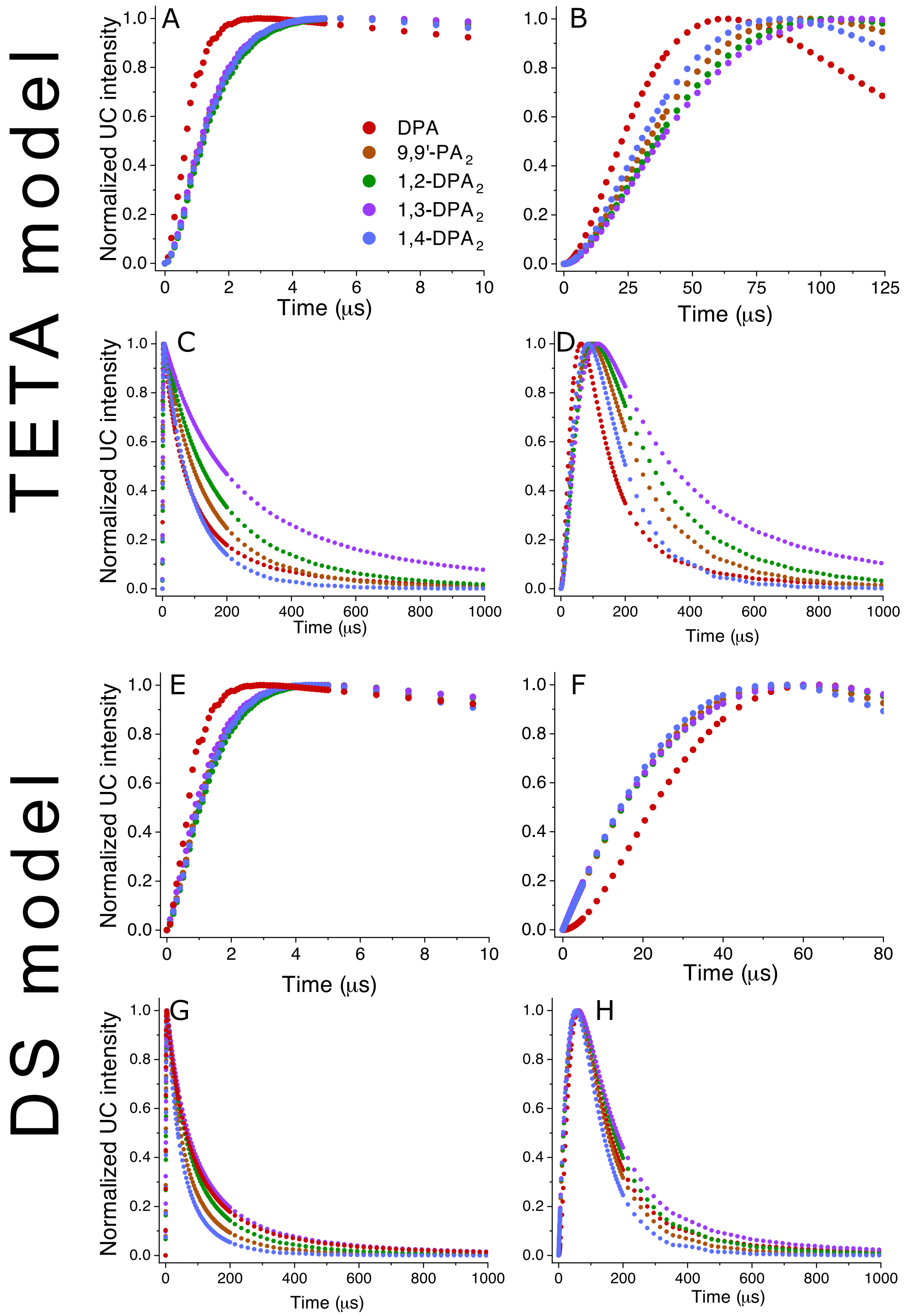

Figure S32. Kinetic simulation of upconverted emission according to the (A-D) TETA model, or (E-H) DS model. Left panel give the traces for high $[^1A]_0/[^1S]_0$ ratios (i.e. 5 μM PtOEP and 1 mM DPA/dimer), and the right panel for low $[^1A]_0/[^1S]_0$ ratios (i.e. 100 μM PtOEP and 10 μM DPA/dimer).

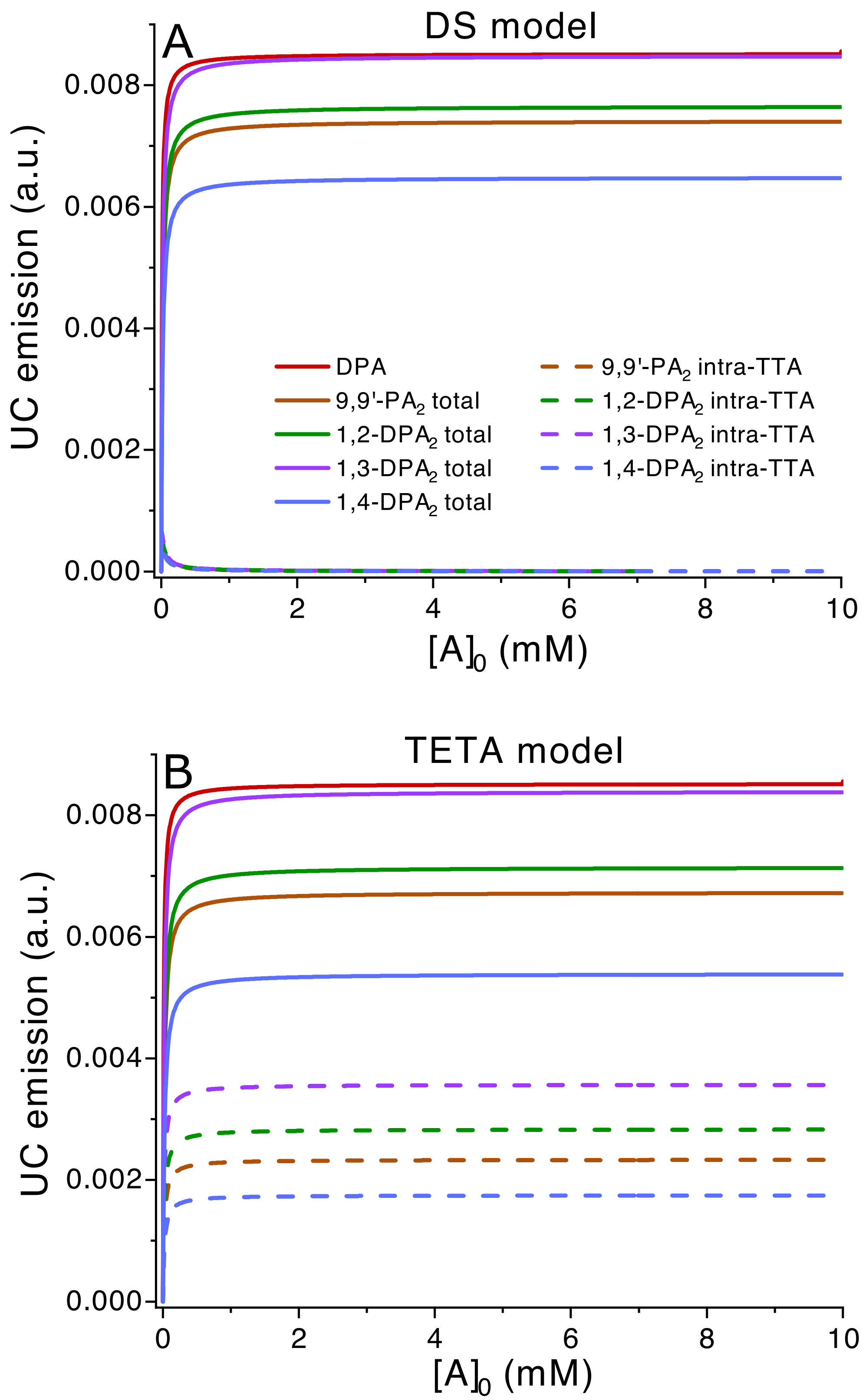

Figure S33. Steady-state simulation of the UC emission intensity for a solution of 5 μM PtOEP and varying initial DPA/dimer concentration following 532 nm cw excitation according to the (A) DS model, and (B) TETA model.

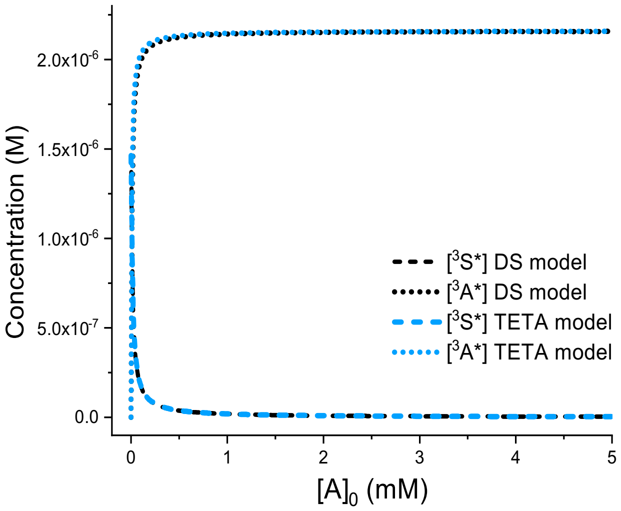

Figure S34. Steady-state simulation of the triplet excited state concentrations for a solution of 5 μM PtOEP and varying initial DPA/dimer concentration following 532 nm cw excitation.

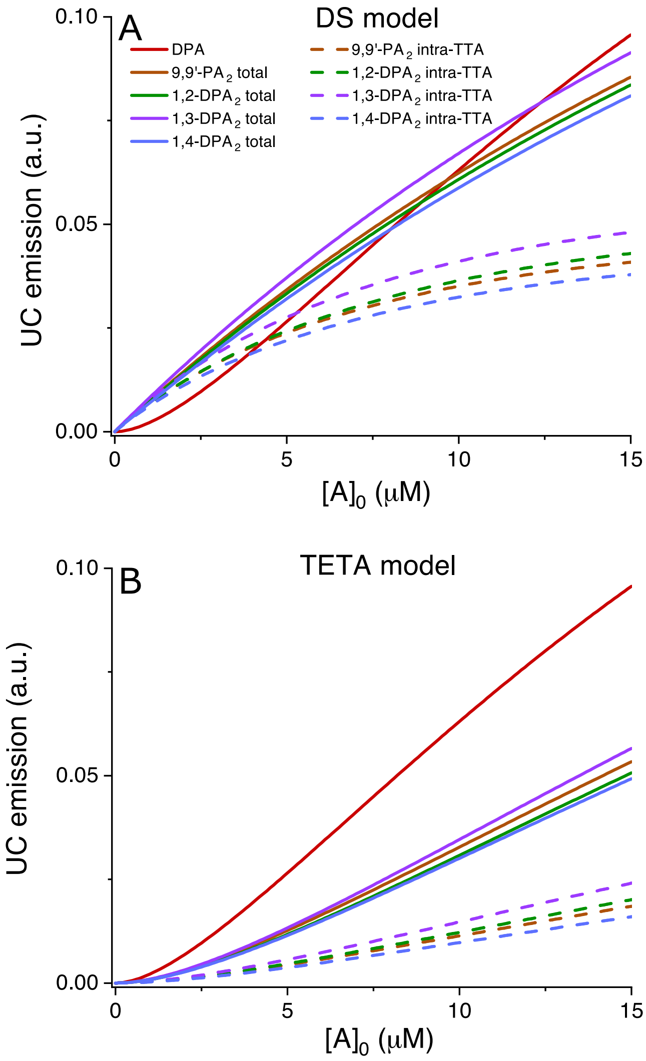

Figure S35. Steady-state simulation of the UC emission intensity for a solution of 100 μM PtOEP and varying initial DPA/dimer concentration following 532 nm cw excitation according to the (A) DS model, and (B) TETA model.

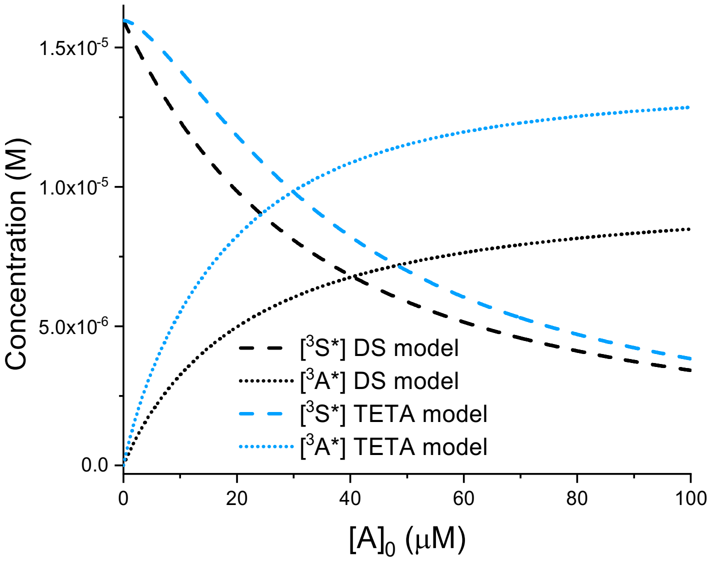

Figure S36. Steady-state simulation of the triplet excited state concentrations for a solution of 100 μM PtOEP and varying initial DPA/dimer concentration following 532 nm cw excitation. Note that in this range of $[A]_0$, the $[^3S^*]$ remains at a significant level.

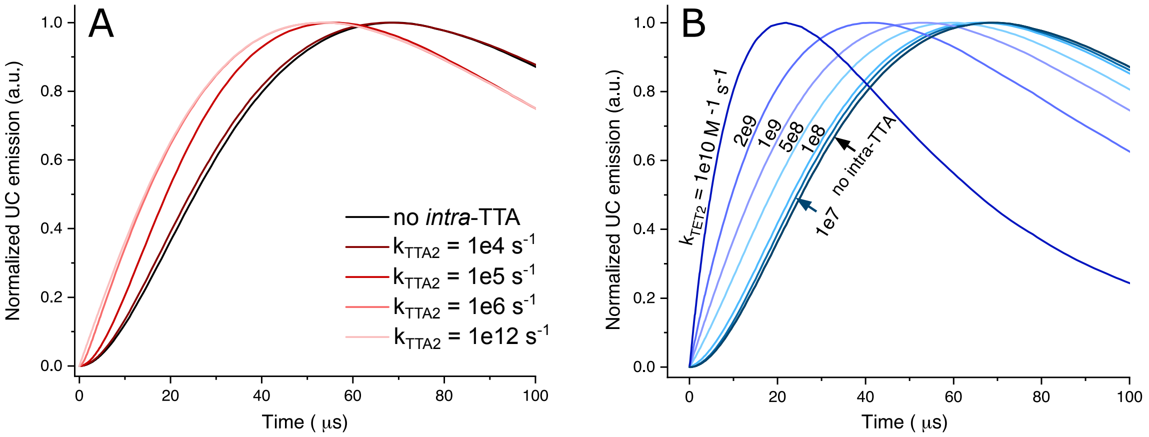

Figure S37. (A) Simulated kinetics of UC emission from 1,4-DPA$_2$ for different values of k_{TTA2}; $k_{TET2} = k_{TET1} = 0.96 \times 10^9$ M^{-1} s^{-1}. The kinetics converge at $k_{TTA2} \approx 10^6$ s^{-1}, which is far below the expected rate of k_{TTA2}. (B) Simulated kinetics of UC emission from 1,4-DPA$_2$ for different values of k_{TET2}; $k_{TTA2} = 10^{12}$ s^{-1}. At expected k_{TET2} rates ($\approx 10^9$ M^{-1} s^{-1}) even small changes in k_{TET2} significantly affects the kinetics.

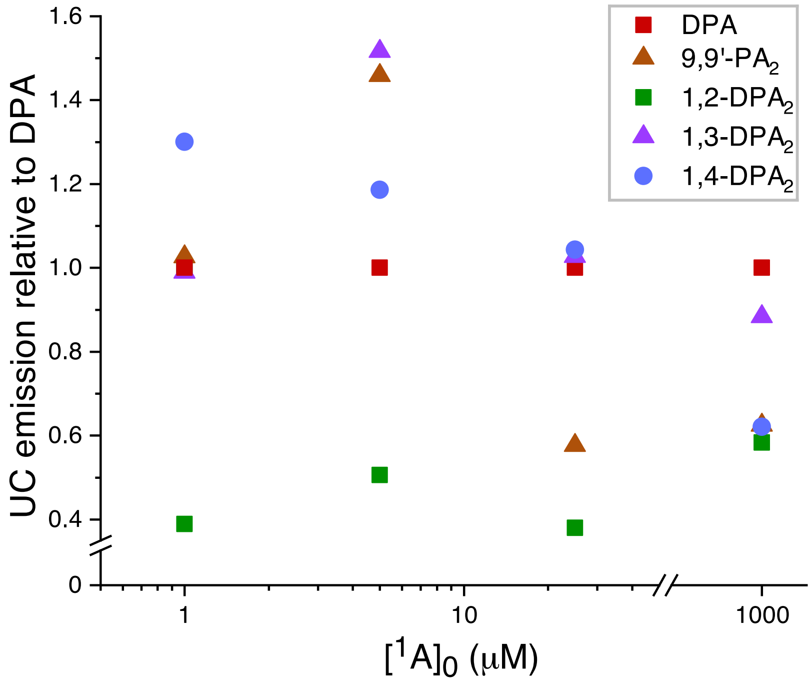

Figure S38. Steady-state UC fluorescence from samples of 100 μM [^{1}S]$_0$ and varying amounts of [^{1}A]$_0$. The rightmost point is in the presence of 6.6 μM [^{1}S]$_0$ (typical UC conditions). The sample concentrations have been adjusted to have the same TET efficiencies for each set of concentrations but are presented in vertical alignment for clarity.

6. References

1. Börjesson, K., Gilbert, M., Dzebo, D., Albinsson, B. & Moth-Poulsen, K. Conjugated anthracene dendrimers with monomer-like fluorescence. *RSC Adv.* **4**, 19846–19850 (2014).
2. Natarajan, P. & Schmittel, M. Photoluminescence, redox properties, and electrogenerated chemiluminescence of twisted 9,9′-bianthryls. *J. Org. Chem.* **78**, 10383–10394 (2013).
3. Nishiuchi, T., Uno, S., Hirao, Y. & Kubo, T. Intramolecular Interaction, Photoisomerization, and Mechanical C–C Bond Dissociation of 1,2-Di(9-anthryl)benzene and Its Photoisomer: A Fundamental Moiety of Anthracene-Based π-Cluster Molecules. *J. Org. Chem.* **81**, 2106–2112 (2016).
4. An, Y. *et al.* Synthesis of Novel Blue Light Emitting 9,9′-bianthracene Derivatives and Their Optoelectronic Properties. *Chinese J. Lumin.* **32**, 890–895 (2011).
5. Heinrich, G., Schoof, S. & Gusten, H. 9,10-diphenylanthracene as a fluorescence quantum yield standard. *J. Photochem.* **3**, 315–320 (1974).
6. Frisch, M. J. *et al.* Gaussian 16, Revision C.01e. (2016).
7. Zhou, Y., Castellano, F. N., Schmidt, T. W. & Hanson, K. On the Quantum Yield of Photon Upconversion via Triplet–Triplet Annihilation. *ACS Energy Lett.* **5**, 2322–2326 (2020).
8. Gray, V. *et al.* Photophysical characterization of the 9,10-disubstituted anthracene chromophore and its applications in triplet-triplet annihilation photon upconversion. *J. Mater. Chem. C* **3**, 11111–11121 (2015).
9. Gray, V. *et al.* Loss channels in triplet-triplet annihilation photon upconversion: Importance of annihilator singlet and triplet surface shapes. *Phys. Chem. Chem. Phys.* **19**, 10931–10939 (2017).
10. Hsu, C. P. The electronic couplings in electron transfer and excitation energy transfer. *Acc. Chem. Res.* **42**, 509–518 (2009).
